# Magmatic tempo of Earth’s youngest exposed plutons as revealed by detrital zircon U-Pb geochronology

**DOI:** 10.1038/s41598-017-12790-w

**Published:** 2017-09-29

**Authors:** Hisatoshi Ito, Christopher J. Spencer, Martin Danišík, Carl W. Hoiland

**Affiliations:** 1 Geosphere Science Sector, Central Research Institute of Electric Power Industry, Chiba, 270-1194 Japan; 2 Earth Dynamics Research Group, Department of Applied Geology, The Institute of Geoscience Research (TIGeR), Curtin University, Perth, WA 6845 Australia; 3 John de Laeter Centre, Department of Applied Geology, The Institute of Geoscience Research (TIGeR), Curtin University, Perth, WA 6845 Australia; 4 Department of Geological Sciences, Stanford University, Stanford, CA 94305 USA

## Abstract

Plutons are formed by protracted crystallization of magma bodies several kilometers deep within the crust. The temporal frequency (i.e. episodicity or ‘tempo’) of pluton formation is often poorly constrained as timescales of pluton formation are largely variable and may be difficult to resolve by traditional dating methods. The Hida Mountain Range of central Japan hosts the youngest exposed plutons on Earth and provides a unique opportunity to assess the temporal and spatial characteristics of pluton emplacement at high temporal resolution. Here we apply U-Pb geochronology to zircon from the Quaternary Kurobegawa Granite and Takidani Granodiorite in the Hida Mountain Range, and from modern river sediments whose fluvial catchments include these plutons in order to reconstruct their formation. The U-Pb data demonstrate that the Kurobegawa pluton experienced two magmatic pulses at ~2.3 Ma and ~0.9 Ma; whereas, to the south, the Takidani pluton experienced only one magmatic pulse at ~1.6 Ma. These data imply that each of these magmatic systems were both spatially and temporally distinct. The apparent ~0.7 Myr age gap between each of the three magmatic pulses potentially constrains the recharge duration of a single pluton within a larger arc plutonic complex.

## Introduction

**Figure 1:**
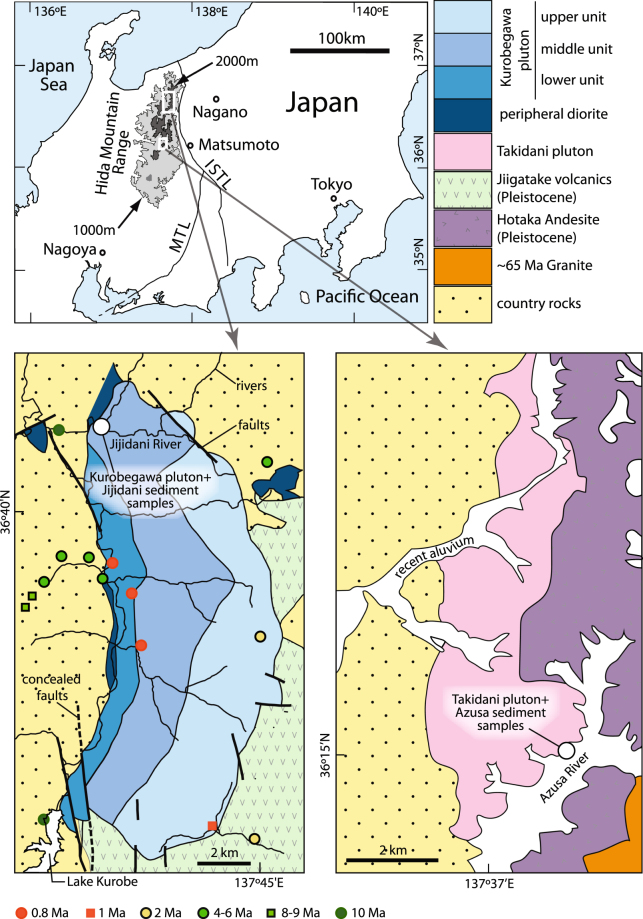
Simplified map of the Hida Mountain Range (HMR) with the sampling locations. Top panel: Location of the HMR on the island of Honshu, Japan. Bottom panels: simplified geological maps of Kurobegawa pluton modified after Wada *et al*.^22^ and Takidani pluton^20,45^. In the Kurobegawa pluton panel, U-Pb ages^3^ are shown and country rocks include pre-Quaternary granitic rocks. ISTL: Itoigawa-Shizuoka Tectonic Line, MTL: Median Tectonic Line. Maps were produced using Adobe Illustrator CC 2014 release (http://blogs.adobe.com/adobeillustrator/tag/2014-release).

Zircon U-Pb geochronology is now widely used to decipher Earth’s evolution from the Hadean to the Quaternary^1–3^. Here we apply this methodology to further constrain the magmatic evolution of the Earth’s youngest exposed plutons, which reside in the Hida Mountain Range (HMR) of central Japan (Fig. 1). In the northern HMR the Kurobegawa Granite has yielded zircon U-Pb ages of ~0.8 Ma, making it Earth’s youngest known pluton^3^. In the central HMR the Takidani Granodiorite yielded a zircon U-Pb age of ~1.4 Ma^4^ (uncorrected for ^230^Th disequilibrium).

As single plutons can crystallize over a protracted period of time^5–9^, a single hand sample may miss the full crystallization history of an entire pluton. Detrital zircon from modern river sediments offers an alternative method from which zircon from an entire plutonic complex can be more accurately represented^10–12^. In order to better understand the dynamics of the Earth’s youngest plutons, we apply zircon U-Pb geochronology to both bedrock and detrital zircon from these plutons sampled from modern river sediments. We employ laser ablation-inductively coupled plasma-mass spectrometry (LA-ICP-MS)^13,14^ because of its high throughput and adequate precision although isotope dilution thermal ionization mass spectrometry (ID-TIMS) has much higher precision and accuracy than LA-ICP-MS^15^. We posit that the results presented herein constrain the timescale and spatial extent of individual plutonic systems and provide insight into the nature and tempo of subduction zone magmatism.

## Geology and samples

The HMR is one of the highest mountain ranges in Japan and is composed primarily of Late Cretaceous to Early Cenozoic plutonic rocks and Pleistocene to Holocene volcanic rocks^16^. The HMR lies just to the west of the Itoigawa-Shizuoka Tectonic Line (i.e. the Eurasian-North American plate boundary), hosts a high density of conjugate reverse fault systems, and is recognized as one of the most active seismotectonic regions of the Japanese islands^17,18^. Exhumation within the HMR is achieved via a combination of structural and surficial denudation processes^19–21^.

The Kurobegawa Granite is a plutonic complex exposed over an area of ~100 km^2^ with vertical exposure from 700 m to 2900 m in elevation in the northern HMR. Although it is broadly referred to as a Neogene granite of 10–0.8 Ma^3^, we follow a more common definition as a Quaternary granite^22^. The Kurobegawa Granite is divided into three units (upper, middle, and lower) based on its geochemistry and texture^22^ and is intrusive into the Jiigatake Volcanic Rocks at its southeastern border, forming a volcanic-plutonic complex^16^. At its southeastern border, the granite becomes leucocratic and contact-metamorphism is observable on the Jiigatake Volcanic Rocks^22,23^. The Jiigatake Volcanic Rocks are caldera-fill volcanic rocks of Quaternary age that extend 16 km in the north-south direction and are locally >3500 m in thickness^24^.

Sampling was performed in the middle unit of the northern rim of the Kurobegawa Granite (Fig. 1). At the sampling site, densely concentrated mafic microgranular enclaves (MMEs) are present and so samples were collected from both the mafic and felsic part of the pluton and analyzed separately. River sands were also collected at the mouth of the west-flowing Jijidani River. Based on geologic mapping^22^ the catchment of the Jijidani River contains the middle and upper units of the Kurobegawa Granite (Fig. 1).

The Takidani Granodiorite is an elongated pluton (13 km × 4 km) with vertical exposure from 1450 m to 2670 m in elevation situated along a major axis of the central HMR (Fig. 1). The pluton is vertically zoned from equigranular biotite-hornblende granodiorite in the deepest through hornblende-biotite granodiorite to hornblende-bearing porphyritic biotite granite at the shallowest level and tilts toward the east^20,25^. It intruded Paleozoic greenstones, a Jurassic accretionary complex, and late Mesozoic to early Cenozoic plutons on its western contact and the Early Pleistocene Hotaka Andesite on the east^25^. The Hotaka Andesite and the Takidani Granodiorite were formed as a volcanic-plutonic complex with close genetic relations^20^. The Hotaka Andesite is composed chiefly of an andesitic to dacitic welded-tuff sheet locally 1500 m in thickness, and subordinate lavas, collapse breccias, diorite porphyry sheets, and a granophyre dike, filling parts of an 18 km × 6 km caldera^20^. The andesitic to dacitic welded-tuff sheet has been metamorphosed to hornblende hornfels within ~800 m, and to biotite hornfels between 800–1500 m from the western contact with the Takidani Granodiorite^20^.

A sample was collected on the southeastern margin of the pluton at its shallowest level. Fluvial sediment of the Azusa River was collected within 20 m distance of the plutonic *in situ* sample. The Azusa River catchment encompasses portions of the Takidani Granodiorite, Hotaka Andesite and early Cenozoic to late Mesozoic plutons (shown as ~65 Ma Granite in Fig. 1) that are exposed to the east of the Hotaka Andesite.

## Results

### Takidani Granodiorite and Azusa River

**Figure 2:**
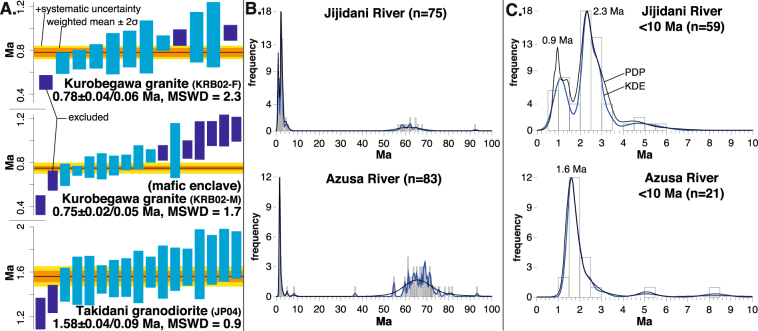
(**A**) Weighted mean zircon ^238^U/^206^Pb ages for the Kurobegawa Granite (top), a mafic enclave from the Kurobegawa Granite (middle), and Takidani Granodiorite (bottom). Purple boxes are excluded analyses. Weighted mean ages are shown with 2σ uncertainty and include analytical errors and systematic errors, respectively. (**B**,**C**) Zircon ^238^U/^206^Pb age distributions (histograms, probability density plot (PDP), and kernel density estimation (KDE)) for detrital zircon from the Jijidani and Azusa river sand younger than 100 Ma (**B**) and 10 Ma (**C**). n = number of grains. Bandwidth for KDE is fixed at 0.25 Ma.

Zircon from the Takidani Granodiorite yielded an age of 1.58 ± 0.09 Ma (2σ, including systematic error) (Fig. 2A). This is consistent with the initial ^230^Th disequilibrium uncorrected U-Pb zircon age of 1.36 ± 0.23 Ma^4^ as ^230^Th correction would shift the age ~0.1 Ma older than the reported age. It is noteworthy that the U-Pb ages of both the shallow (this study) and deep^4,26^ levels of pluton emplacement showed nearly coeval age of ~1.6–1.5 Ma. Detrital zircon from the Azusa River show two age groups: ages with a peak of ~65 Ma and ages less than 10 Ma (Fig. 2B). The age group of <10 Ma has a prominent peak of ~1.6 Ma (Fig. 2C), which is in agreement with the age of the Takidani Granodiorite.

Chemically etched zircon grains revealed a unique feature (Supplementary Fig. S2). As is expected, Quaternary zircon has a low spontaneous fission-track density (Fig. S2A). The ~65 Ma zircons have two groups: those with low (Fig. S2B) and high (Fig. S2C) track densities. The ~65 Ma zircons with a low track density are interpreted as annealed xenocrystic zircons incorporated in the Takidani Granodiorite or Hotaka Andesite magmas whereas those with a high track density should be derived from the outcropping ~65 Ma granitic rocks.

### Kurobegawa Granite and Jijidani River

Zircon from the Kurobegawa Granite yielded 0.78 ± 0.06 Ma for the felsic part and 0.75 ± 0.05 Ma for a mafic enclave (Fig. 2A). Therefore both the felsic and mafic parts of the granite formed almost coevally, consistent with evidence for local magma mingling^22^. These ages are consistent with those obtained previously^3^ for the lower and middle units of the Kurobegawa Granite further to the south (Fig. 1), reaffirming that the Kurobegawa Granite is the Earth’s youngest known exposed granitoid pluton.

Detrital zircons from the Jijidani River show two age groups: ages with a small peak of ~65 Ma and ages with a large peak of less than 10 Ma (Fig. 2B). The age group of <10 Ma has two major peaks of ~0.9 Ma and ~2.3 Ma (Fig. 2C). This contrasts with the age distribution of zircon from the Azusa River, which has a single age population of ~1.6 Ma. We interpret these data as evidence that the Kurobegawa Granite experienced two intrusion episodes of ~0.9 Ma and ~2.3 Ma in contrast to the single ~1.6 Ma Takidani Granodiorite and Hotaka Andesite magmatism. Ref.^3^ reported age peaks of ~0.8 Ma and ~2.3 Ma for the Quaternary Kurobegawa Granite from the southern half of the pluton (Fig. 1).

## Discussion

The data presented in this study are the youngest U-Pb zircon ages of any plutonic rock on Earth. The prodigious exhumation rates of the Japanese Alps provide a unique opportunity to probe the development of the Earth’s youngest known exposed pluton. In the case of the Takidani Granodiorite, the plutonic complex is genetically and temporally associated with the correlative volcanic unit of the Hotaka Andesite^20^. A caldera-forming eruption of the Hotaka Andesite with an ~1.7 Ma eruption age of the outflow facies (Nyukawa Pyroclastic Flow Deposit) and correlated widespread tephra (Ho-Kd 39 Tephra) along with paleomagnetic and biostratigraphic studies^27^ support this volcanic-plutonic relationship. Furthermore, the near unimodal <10 Ma detrital zircon age peak from the Azusa River implies the Hotaka-Takidani volcanic-plutonic complex was formed within a single relatively contracted period of time.

In contrast to the Takidani plutonic complex, the modern river draining the northern part of the Kurobegawa plutonic complex exhibits detrital zircon age spectra with two prominent age peaks at ~0.9 Ma and ~2.3 Ma with bedrock ages indistinguishable from the younger age peak. The ages of the felsic and mafic phases of the Kurobegawa Granite are within uncertainty of analysis and therefore cannot resolve relative timing. A petrologic assessment^22^ argues that mafic magma intruded into the host felsic magma, eventually forming MMEs.

The Kurobegawa Granite and its surroundings host a great number of hot springs and geothermal activity^28^. Its youngest intrusion (~0.8 Ma) is considerably younger than the Geysers Plutonic complex (1.8–1.1 Ma^29^), which is the assumed heat source for the largest economically viable geothermal field on the planet. Due to its extremely elevated geothermal gradient^30^ and thus shallow brittle-ductile transition we posit that the Kurobegawa Granite may also provide economically viable geothermal energy using supercritical water^31^.

It has been proposed that a hierarchy of magmatic tempos is present at the various scales of volcanic arcs, volcanic-plutonic systems, individual plutons, caldera forming eruptions, to smaller near-steady state eruptions^5,32,33^. If plutonic tempo can be approximated as average pluton volume divided by magmatic flux, then since pluton size is a function of several independent variables (e.g. composition, rheology, stress, and geothermal gradient) plutonic tempo is expected to vary widely between geologic settings and locales. The Quaternary plutonic complexes of the HMR – for which most of these variables are known – thus provide an important measure against which to test geophysical modeling of the mechanisms and timescales of pluton formation.

Magmatic episodicity on the <1 Myr timescale described above has never before been documented in plutonic systems. Volcanic systems, however, do often exhibit episodic behavior on similar timescales with protracted zircon growth and marked periods of magmatic quiescence and form in spatially distinct patterns. For example, significant volcanic pulses separated by hundreds of thousands of years have been documented in the Central Andes^34,35^, Yellowstone^36,37^, Taupo^38^, and Aegean Sea^13^. The ~0.7 Myr hiatus of the HMR is unusual for plutonic rocks and implies the temporal hierarchy of individual plutonic systems is faster than previously thought. It should further be noted that as zircon crystallization is directly connected to zircon saturation, the zircon record provides a minimum estimate to the longevity of a magmatic system. The Quaternary plutons of the HMR provide unique insight into the hierarchy of magmatic tempo and highlight the need to examine these timescales within young plutonic systems wherein higher temporal resolution is achievable.

## Methods

Zircon grains were extracted by conventional heavy liquid and magnetic separations, handpicked, embedded in a PFA Teflon sheet and polished in a similar fashion to fission-track studies^39,40^. LA-ICP-MS was employed to determine U-Pb ages in zircon (see *supplementary information* for complete description of methods). An initial assessment of the U-Pb ages revealed that a large proportion (>60%) were older than 10 Ma for the Azusa River sample. Therefore, to preferentially analyze young zircon associated with the Quaternary plutons, grains were etched in NaOH and KOH eutectic melt at ~210 °C for up to 20 hours to reveal spontaneous fission-tracks (Fig. S2). Zircon grains with low spontaneous fission-track density were mainly selected for further analyses as these were expected for Quaternary zircon^40^. Some grains with high spontaneous track density were intentionally selected so as to date old zircon grains (JP3-2-101–JP3-2-110 in Table S2). U-Pb ages were obtained following procedures described in ref.^14^ with LA-ICP-MS equipment (a Thermo Fisher Scientific ELEMENT XR magnetic sector-field ICP-MS coupled to a New Wave Research UP-213 Nd-YAG laser) at the Central Research Institute of Electric Power Industry. All age data are shown in Table S2. Note that etching does not affect U-Pb ages as is demonstrated that the same age (~33.5 Ma) was obtained for secondary reference zircon of OD-3 (ref.^41^) both for unetched and 13-hour-etched zircon (Table S2). Initial ^230^Th disequilibrium correction was done assuming the (Th/U)_magma_ was 5.0, which is essentially the same with the value of 4.8 that was employed in ref.^3^ for dating the Kurobegawa Granite. A systematic uncertainty of 5% was added quadratically to the weighted average uncertainty to account for the excess dispersion in the reference materials. Weighted means and reduced chi-squared statistics were calculated using KD*Χ* and assessed for over- and under-dispersion^42,43^. Age spectra were visualized using DensityPlotter^44^.

## Electronic supplementary material

Supplementary information
