# Supplementary material for: Magmatic tempo of Earth’s youngest exposed plutons as revealed by detrital zircon U-Pb geochronology
Source: Sci Rep. 2017 Sep 29;7:12457. doi: 10.1038/s41598-017-12790-w (PMC5622053; doi:10.1038/s41598-017-12790-w)
Supplement: Supplementary file 1 — Supplementary information [file 41598_2017_12790_MOESM1_ESM.pdf]

Magmatic tempo of Earth's youngest exposed plutons as revealed by detrital zircon U-Pb geochronology

Supplementary information

Hisatoshi Ito, Christopher Spencer, Martin Danišík, Carl Hoiland

U-Pb method

LA-ICP-MS U-Pb dating was performed at the Central Research Institute of Electric Power Industry, using experimental conditions shown in Table S1. Samples were ablated in helium gas by pulses at a 10 Hz repetition rate. The focus of the laser beam was fixed at the sample surface throughout the data acquisition. Two different sets of laser ablation parameters were adopted: 1) 40 μm laser spot with 3–4 J/cm^2 energy density, and 2) 30 μm laser spot with 7–8 J/cm^2 energy density. The reason for changing experimental parameters (from 40 μm spot size to 30 μm) was simply that the former parameter was adopted in 2013 and the latter one was adopted since 2016. Data were acquired in electrostatic scanning (E-scan) mode over 1080 mass scans during a 30 s background measurement, followed by a 30 s sample ablation and then a 45 s background (washout) measurement. ^{235}U was calculated from ^{238}U assuming ^{238}U/^{235}U = 137.818 (ref. 1). Both unknowns and standards were measured in the same conditions.

Data for the first 10 s of ablation were neglected to avoid surface Pb contamination and signal instability, and the following 10 s of data were used for age calculation. Approximately ~18 μm with 3–4 J/cm^2 energy density (~24μm with 7–8 J/cm^2 energy density) were drilled during the 30 s laser ablation and therefore data from 6–12 μm (8–16 μm) depths were analyzed.

Raw data were processed offline using an Excel spreadsheet program created by the first author. After gas blank correction, laser-induced elemental fractionation and instrumental mass discrimination for ^{207}Pb/^{235}U and ^{206}Pb/^{238}U ratios were corrected by normalization to the Fish Canyon Tuff (FCT) zircon[2]. The drift of the Pb/U ratio during the analytical session was monitored and corrected by the NIST SRM 610 glass standard, which was analyzed every 12 (in-between 10 unknowns, two standard zircons of Plesovice[3] and 91500 (ref. 4) ablations). Note that NIST SRM 612 was used instead of 91500 in the 2013 experiments. No down-hole isotope ratios (Pb/U, Th/U) fractionation correction[5] was performed because data from the same spot size and depth range were used for standards and unknowns in each experiment. U and Th concentrations were quantified by comparing counts of ^{238}U and ^{232}Th for the sample relative to the standard 91500, which is assumed to have homogeneous U and Th concentrations of 80 and 30 ppm respectively[4, 6].

FCT zircon was used as a U-Pb reference material because it has been precisely dated by the ID-TIMS U-Pb method[2, 7] and our repeated analyses of FCT confirmed its suitability as a U-Pb reference zircon[8-11]. FCT zircons show a wide range of U and Th concentration and Th/U ratio[2], whereas the standard 91500 zircons show a very narrow range. Therefore, for these values 91500 data were used instead of FCT data. Note that the U and Th contents for 40μm laser beam were estimated using 610 glass standard because we did not possess 91500 standard in 2013. To test the validity of the applied method and the reproducibility of the obtained age data, zircons of known age from the OD-3 (33.0 ± 0.1 Ma[12]) together with Plesovice (337.13 ± 0.37 Ma[3]) were also dated during the analytical session. The concordance of the obtained ages and their reference ages assures that the analytical conditions are valid.

Individual $^{206}Pb/^{238}U$ grain ages were determined using the $^{206}Pb/^{238}U$ ratio, and the error was calculated based on the time-series fluctuation (standard error) of $^{206}Pb/^{238}U$ for the time range adopted (i.e., 40–50 s from the start of the analysis).

Young (e.g., <2 Ma) U-Pb zircon ages are strongly affected by disequilibrium of ^{230}Th at the time of zircon crystallization from the magma[13]. Therefore, individual zircon U-Pb ages were corrected using corrected $^{206}Pb/^{238}U$ ratio ($^{206}Pb/^{238}U_{corrected}$) as follows using a factor f, where $f = (Th/U)_{zircon}/(Th/U)_{magma}$:

$$^{206}Pb/^{238}U_{corrected} = {}^{206}Pb/^{238}U_{measured} + (\lambda_{238}/\lambda_{230}) \times (1 - f)$$

where $^{206}Pb/^{238}U_{measured}$ is a measured $^{206}Pb/^{238}U$ ratio, λ_{238} of 1.55125×10^{-10} yr^{-1} and λ_{230} of 0.922×10^{-5} yr^{-1} are decay constants of ^{238}U and ^{230}Th, respectively. Recently, Ref. 14 argued that Ref. 13's methodology is inappropriate for ages <0.4 Ma and proposed a more robust methodology. Therefore care should be taken for ages <0.4 Ma. In this study, all accepted ages are >0.4 Ma (Fig. 2; Table S2).

Common-lead correction was done using the ^{207}Pb method[15, 16], in which the present $^{207}Pb/^{206}Pb$ production ratio of 0.0461 and the common $^{207}Pb/^{206}Pb$ ratio of 0.832 (ref. 16) were adopted without uncertainties. The percentage of common ^{206}Pb ($f_{206}\%$) is obtained as follows:

$$f_{206}\% = 100 \times (x - 0.0461)/(0.832 - 0.0461)$$

where x is a measured $^{207}Pb/^{206}Pb$ ratio. Using $f_{206}\%$, the common-lead corrected $^{206}Pb/^{238}U$ ratio ($^{206}Pb/^{238}U_{c\text{-}corrected}$) or radiogenic $^{206}Pb/^{238}U$ ratio is calculated as follows:

$$^{206}Pb/^{238}U_{c\text{-}corrected} = (1 - f_{206}\%/100) \times {}^{206}Pb/^{238}U$$

where $^{206}Pb/^{238}U$ is a measured $^{206}Pb/^{238}U$ ratio for age standards and ^{230}Th-corrected $^{206}Pb/^{238}U$ ratio for unknown samples. The uncertainty of radiogenic $^{206}Pb/^{238}U$ was kept the same with that of the measured $^{206}Pb/^{238}U$.

In case raw data were significantly disturbed by a single abnormal pulse, the pulse data were substituted by the average of the former and the latter pulses data as shown in Fig. S1. These data were affixed as (2) after the sample code (Table S2).

Data with both <75% for $f_{206}\%$ and <30% age uncertainty were adopted.

Tera-Wasserburg plot

As mentioned above, the U-Pb age was calculated assuming the common $^{207}Pb/^{206}Pb$ ratio of 0.832. Figure S3 shows ages without the assumption of common $^{207}Pb/^{206}Pb$ ratio of 0.832 using the Tera-Wasserburg plot[17]. As for the Kurobegawa Granite, the Tera-Wasserburg concordia age yields 0.90 ± 0.06 Ma for felsic part and 0.96 ± 0.17 Ma for mafic enclave. These ages are ~0.1 Ma older than the ages assuming the common $^{207}Pb/^{206}Pb$ ratio of 0.832. As for the Takidani Granodiorite, the Tera-Wasserburg concordia age yields 1.52 ± 0.08 Ma, which is in agreement with the age of 1.58 ± 0.09 Ma assuming the common $^{207}Pb/^{206}Pb$ ratio of 0.832. As for the Azusa River sand, the Tera-Wasserburg concordia age yields 1.63 ± 0.20 Ma, which is in agreement with the peak age of ~1.6 Ma. Overall, the discrepancy between ages assuming the common $^{207}Pb/^{206}Pb$ ratio of 0.832 and ages without the assumption of common $^{207}Pb/^{206}Pb$ ratio of 0.832 are small, and we assume the conclusions are the same in both cases. In Fig. S3C, the y-axis intercept of the regression line is 0.736, which may indicate that the common $^{207}Pb/^{206}Pb$ ratio of 0.736 is more appropriate than the value of 0.832. Nevertheless, we assume the choice of common $^{207}Pb/^{206}Pb$ ratio does not affect our conclusions.

References

1. Hiess, J., Condon, D.J., McLean, N. & Noble, S.R. $^{238}U/^{235}U$ systematics in terrestrial U-bearing minerals. *Science* **335**, 1610–1614 (2012).
2. Schmitz, M.D. & Bowring, S.A. U-Pb zircon and titanite systematics of the Fish Canyon Tuff: an assessment of high-precision U-Pb geochronology and its application to young volcanic rocks. *Geochim. Cosmochim. Acta* **65**, 2571–2587 (2001).
3. Sláma, J. *et al.* Plesovice zircon — A new natural reference material for U-Pb and Hf isotopic microanalysis. *Chem. Geol.* **249**, 1–35 (2008).
4. Wiedenbeck, M. *et al.* Further characterisation of the 91500 zircon crystal. *Geostandards Geoanalytical Res.* **28**, 9–39 (2004).
5. Paton, C *et al.* Improved laser ablation U-Pb zircon geochronology through robust downhole fractionation correction. *Geochem. Geophys. Geosyst.* **11**, Q0AA06 (2010).
6. Wiedenbeck, M. *et al.* Three natural zircon standards for U–Th–Pb, Lu–Hf, trace element and REE analyses. *Geostand. Newslett.* **19**, 1–24 (1995).
7. Wotzlaw, J.-F. *et al.* Tracking the evolution of large-volume silicic magma reservoirs from assembly to supereruption. *Geology* **41**, 867–870 (2013).
8. Ito, H. *et al.* Earth's youngest exposed granite and its tectonic implications: the 10–0.8 Ma Kurobegawa Granite. *Sci. Rep.* **3**, 1306; 10.1038/srep01306 (2013).
9. Ito, H. *et al.* Quaternary plutonic magma activities in the southern Hachimantai

geothermal area (Japan) inferred from zircon LA-ICP-MS U–Th–Pb dating method: *J. Volcanol. Geotherm. Res.* **265**, 1–8 (2013).
10. Ito, H. Zircon U–Th–Pb dating using LA-ICP-MS: Simultaneous U–Pb and U–Th dating on the 0.1 Ma Toya Tephra, Japan. *J. Volcanol. Geotherm. Res.* **289**, 210–223 (2014).
11. Ito, H., Uesawa, S., Nanayama, F. & Nakagawa, S. Zircon U–Pb dating using LA-ICP-MS: Quaternary tephras in Yakushima Island, Japan. *J. Volcanol. Geotherm. Res.* **338**, 92–100 (2017).
12. Iwano, H. *et al.* An inter-laboratory evaluation of OD-3 zircon for use as a secondary U–Pb dating standard. *Island Arc* **22**, 382–394 (2013).
13. Schärer, U. The effect of initial ^{230}Th disequilibrium on young U-Pb ages: The Makalu case, Himalaya. *Earth Planet. Sci. Lett.* **67**, 191–204 (1984).
14. Sakata, S. *et al.* A new approach for constraining the magnitude of initial disequilibrium in Quaternary zircons by coupled uranium and thorium decay series dating. *Quat. Geochronol.* **38**, 1–12 (2017).
15. Williams, I.S. U-Th-Pb geochronology by ion microprobe. In: McKibben, M.A., Shanks III, W.C., Ridley, W.I. (eds.): Applications of Microanalytical Techniques to Understanding Mineralizing Processes. *Reviews in Economic Geology* **7**, pp. 1–35 (1998).
16. Cocherie, A., Fanning, C.M., Jezequel, P. & Robert, M. LA-MC-ICPMS and SHRIMP U–Pb dating of complex zircons from Quaternary tephras from the French Massif Central: Magma residence time and geochemical implications. *Geochim. Cosmochim. Acta* **73**, 1095–1108 (2009).
17. Tera, F. & Wasserburg, G.J. U-Th-Pb systematics in three Apollo 14 basalts and the problem of initial Pb in lunar rocks. *Earth Planet. Sci. Lett.* **14**, 281–304 (1972).

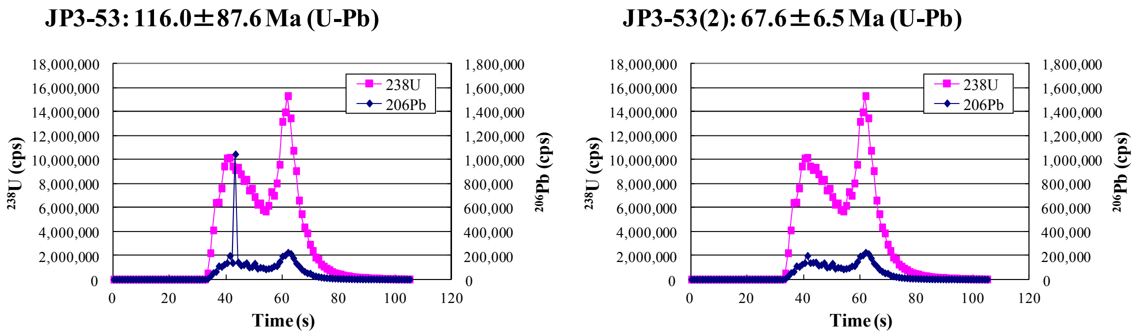

Figure S1. Raw data with an abnormally high pulse (JP3-53) and corresponding data (JP3-53(2)) that substitutes the high pulse data with the average of the former and the latter pulses data. U-Pb ages with 2σ error are shown.

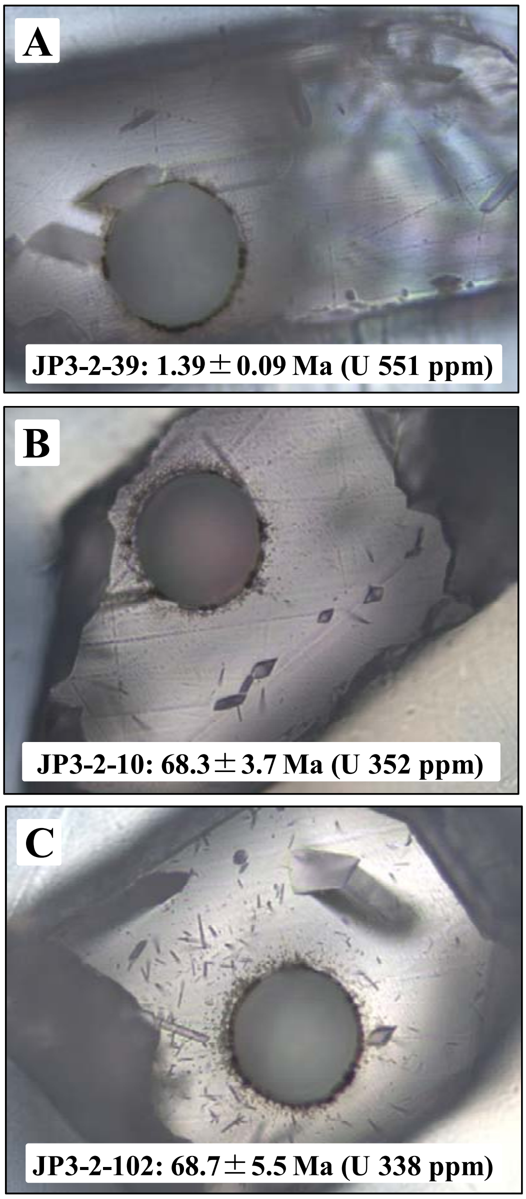

Figure S2. Representative etched zircons from Azusa River sand. U-Pb age with 2σ error and U content (ppm) are shown. Ablation pits are all 30 μm in diameter.

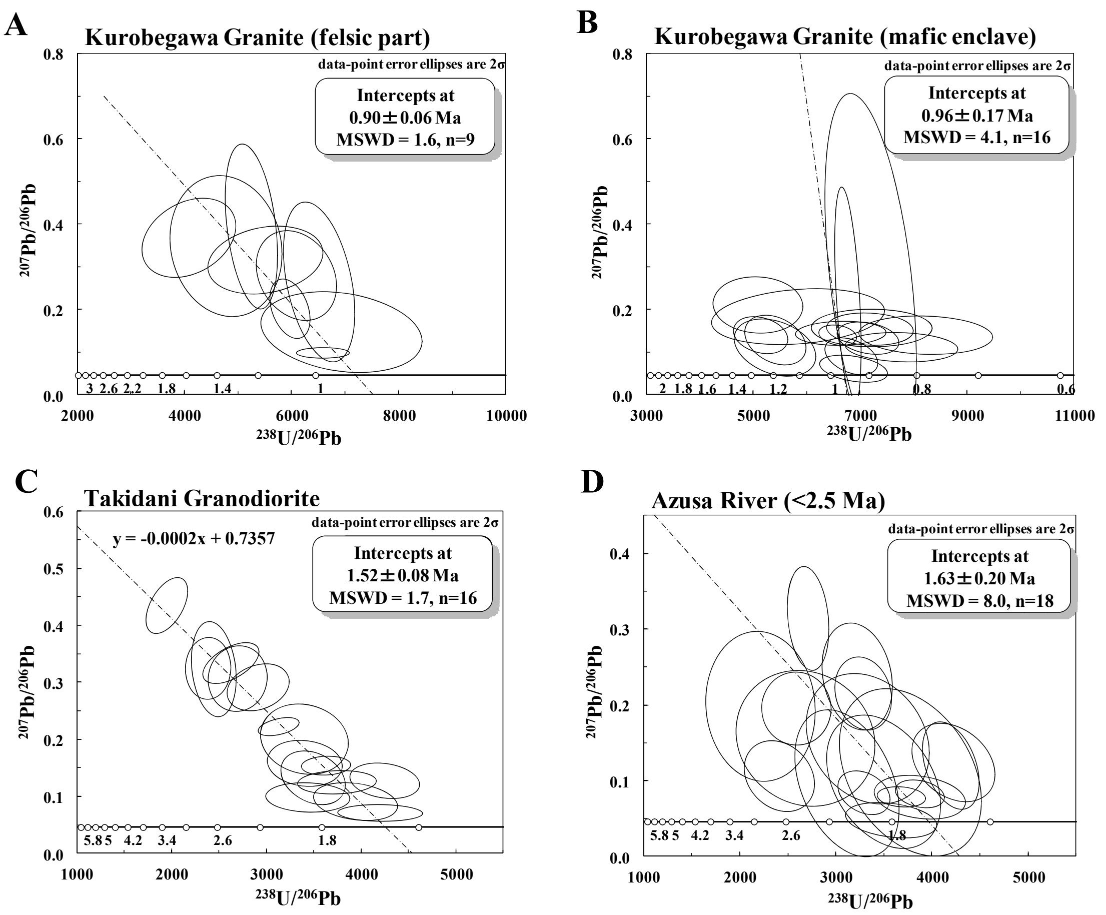

Figure S3. Tera-Wasserburg plot for Kurobegawa Granite (felsic part) (A), Kurobegawa Granite (mafic enclave) (B), Takidani Granodiorite (C), and Azusa River sand (D). U-Pb ages of <2.5 Ma are plotted for Azusa River sand. All ages are corrected for initial ^{230}Th disequilibrium.

Table S1
LA-ICP-MS operating conditions.

**Laboratory & Sample Preparation**	
Laboratory name	Central Research Institute of Electric Power Industry, Japan
Sample type/mineral	Zircons
Sample preparation	Conventional separation, 1 cm PFA Teflon mount, unpolished or 1 µm polish to finish
**Laser ablation system**	
Make, model & type	New Wave Research UP-213
Ablation cell & volume	NWR standard 1-volume cell, volume ~3cm^3
Laser wavelength	213 nm
Pulse width	4 ns
Energy density/fluence	3–4 J/cm^2 for 40 µm laser beam or 7–8 J/cm^2 for 30 µm laser beam
Repetition rate	10 Hz
Ablation duration	30 s
Ablation pit depth	~18 µm with 3–4 J/cm^2 and ~24 µm with 7–8 J/cm^2, measured using confocal laser microscopy
Spot size	30 µm or 40 µm
Sampling mode	Single hole drilling, laser beam focused at the surface
Carrier gas and flow	100% He, 0.5 l/min
**ICP-MS Instrument**	
Make, model & type	Thermo Fisher Scientific ELEMENT XR, SF-ICP-MS
Sample introduction	Ablation aerosol only
RF power	1200 W
Make-up gas flow	0.8 l/min Ar
Detection system	Single detector triple mode
Masses measured	202, 204, 206, 207, 208, 230, 232, 238
Integration time per peak	10 ms
Number of mass scans	1080
Total integration time per reading	~1 s
Detector deadtime	11 ns
**Data Processing**	
Gas blank	30 s prior to each ablation spot
Calibration strategy	Fish Canyon Tuff (FCT) used as primary reference material, Plesovice, 91500 & OD-3 used as secondaries for quality control
Reference material information	FCT ^{206}Pb/^{238}U 0.004421 & ^{207}Pb/^{235}U 0.02867 (Schmitz & Bowring, 2001)
Data processing package used	In-house spreadsheet data processing
Mass discrimination	Mass bias correction normalized to the primary reference material
Common-Pb correction	^{207}Pb method (Williams, 1998)
Uncertainty level & propagation	Ages are quoted at 2σ, propagation is by quadratic addition.
Quality control/validation	Plesovice: Wtd ave. ^{206}Pb/^{238}U age = 337.7 ± 1.1 Ma (2σ),<br>OD-3: Wtd ave. ^{206}Pb/^{238}U age = 33.5 ± 0.5 Ma (2σ)
Other information	Drift of Pb/U ratio corrected by NIST SRM 610 glass standard

Table S2

LA-ICP-MS zircon U-Pb analytical results. Data in italics (>75% common Pb contamination or >30% uncertainty) were excluded for further U-Pb analyses.

Sample name	Condition[a]	Th	U	Th/U	f[b]	f_{206}%[c]	Total						Radiogenic		Age [Ma]		MSWD[d]
		(ppm)	(ppm)				$^{207}Pb/^{206}Pb$	2σ	$^{207}Pb/^{235}U$	2σ	$^{206}Pb/^{238}U$	2σ	$^{206}Pb/^{238}U$	2σ	$^{206}Pb/^{238}U$	2σ	
Jijidani River (sampling location coordinates: 36.698189,137.680128)																	
KRB07-1	40μmPE0	288	785	0.20	0.04	0.5	0.050	0.009	0.0036	0.0006	0.000514	0.000022	0.000511	0.000022	3.30	0.14	
KRB07-2	40μmPE0	611	1,839	0.19	0.04	-2.5	0.027	0.000	0.0529	0.0025	0.014451	0.000591	0.014451	0.000591	92.49	3.81	
KRB07-3	40μmPE0	145	289	0.28	0.06	22.7	0.224	0.075	0.0072	0.0025	0.000231	0.000033	0.000179	0.000033	1.15	0.21	
KRB07-4	40μmPE0	455	1,147	0.22	0.04	0.0	0.046	0.007	0.0029	0.0004	0.000451	0.000021	0.000451	0.000021	2.91	0.14	
KRB07-5	40μmPE0	401	440	0.51	0.10	11.0	0.133	0.029	0.0091	0.0026	0.000498	0.000064	0.000443	0.000064	2.85	0.41	
KRB07-6	40μmPE0	238	389	0.34	0.07	28.4	0.269	0.273	0.0061	0.0054	0.000164	0.000024	0.000117	0.000024	0.76	0.15	
KRB07-7	40μmPE0	57	90	0.35	0.07	51.4	0.450	0.043	0.1097	0.0160	0.001769	0.000179	0.000860	0.000179	5.54	1.15	
KRB07-8	40μmPE0	198	188	0.59	0.12	-0.6	0.041	0.003	0.0582	0.0043	0.010288	0.000363	0.010288	0.000363	65.98	2.34	
KRB07-9	40μmPE0	161	190	0.47	0.09	8.5	0.113	0.036	0.0067	0.0021	0.000430	0.000053	0.000393	0.000053	2.54	0.34	
KRB07-10	*40μmPE0*	*170*	*284*	*0.33*	*0.07*	*30.0*	*0.282*	*0.071*	*0.0583*	*0.0134*	*0.001499*	*0.000466*	*0.001049*	*0.000466*	*6.76*	*3.01*	
KRB07-11	30μmPE0	227	260	0.87	0.17	18.4	0.190	0.073	0.0128	0.0068	0.000486	0.000053	0.000397	0.000053	2.56	0.34	
KRB07-12	30μmPE0	220	538	0.41	0.08	6.4	0.096	0.046	0.0028	0.0014	0.000209	0.000022	0.000196	0.000022	1.26	0.14	
KRB07-13	30μmPE0	553	686	0.81	0.16	6.1	0.094	0.018	0.0048	0.0013	0.000373	0.000025	0.000350	0.000025	2.26	0.16	
KRB07-14	30μmPE0	128	197	0.65	0.13	0.2	0.047	0.004	0.0694	0.0059	0.010610	0.000379	0.010592	0.000379	67.92	2.45	
KRB07-15	30μmPE0	92	206	0.45	0.09	24.6	0.240	0.142	0.0128	0.0084	0.000386	0.000034	0.000291	0.000034	1.88	0.22	
KRB07-16	30μmPE0	852	1,757	0.48	0.10	1.5	0.058	0.010	0.0036	0.0006	0.000455	0.000059	0.000448	0.000059	2.89	0.38	
KRB07-17	*30μmPE0*	*148*	*294*	*0.50*	*0.10*	*63.8*	*0.547*	*0.035*	*0.1133*	*0.0179*	*0.001502*	*0.000203*	*0.000544*	*0.000203*	*3.51*	*1.31*	
KRB07-18	30μmPE0	747	909	0.82	0.16	58.4	0.505	0.106	0.0270	0.0075	0.000387	0.000047	0.000161	0.000047	1.04	0.30	
KRB07-19	30μmPE0	793	824	0.96	0.19	4.9	0.084	0.021	0.0058	0.0014	0.000502	0.000035	0.000478	0.000035	3.08	0.23	
KRB07-20	*30μmPE0*	*315*	*455*	*0.69*	*0.14*	*28.6*	*0.271*	*0.097*	*0.0107*	*0.0109*	*0.000288*	*0.000106*	*0.000206*	*0.000106*	*1.33*	*0.68*	
KRB07-21	30μmPE0	394	683	0.58	0.12	22.2	0.220	0.057	0.0072	0.0018	0.000238	0.000029	0.000185	0.000029	1.19	0.19	
KRB07-22	30μmPE0	1,198	1,006	1.19	0.24	5.1	0.086	0.016	0.0046	0.0007	0.000391	0.000021	0.000371	0.000021	2.39	0.14	
KRB07-23	30μmPE0	487	436	1.12	0.22	11.8	0.139	0.047	0.0050	0.0015	0.000263	0.000020	0.000232	0.000020	1.50	0.13	
KRB07-24	30μmPE0	827	786	1.05	0.21	7.7	0.106	0.026	0.0072	0.0016	0.000488	0.000039	0.000450	0.000039	2.90	0.25	
KRB07-25	*30μmPE0*	*445*	*539*	*0.83*	*0.17*	*67.7*	*0.578*	*0.104*	*0.1975*	*0.1253*	*0.002479*	*0.001245*	*0.000802*	*0.001245*	*5.17*	*8.02*	
KRB07-26	30μmPE0	348	259	1.34	0.27	18.4	0.190	0.112	0.0124	0.0041	0.000472	0.000057	0.000385	0.000057	2.48	0.37	
KRB07-27	30μmPE0	198	190	1.04	0.21	0.5	0.050	0.009	0.0665	0.0117	0.009717	0.000273	0.009673	0.000273	62.05	1.76	
KRB07-28	30μmPE0	1,852	2,534	0.73	0.15	8.0	0.109	0.021	0.0082	0.0017	0.000548	0.000022	0.000504	0.000022	3.25	0.14	
KRB07-29	*30μmPE0*	*290*	*267*	*1.08*	*0.22*	*51.3*	*0.449*	*0.053*	*0.0478*	*0.0148*	*0.000772*	*0.000169*	*0.000376*	*0.000169*	*2.42*	*1.09*	
KRB07-30	30μmPE0	183	322	0.57	0.11	18.8	0.194	0.077	0.0049	0.0011	0.000185	0.000021	0.000150	0.000021	0.97	0.14	
KRB07-31	30μmPE0	207	367	0.56	0.11	39.8	0.359	0.079	0.0113	0.0044	0.000230	0.000036	0.000138	0.000036	0.89	0.23	
KRB07-32	*30μmPE0*	*254*	*388*	*0.66*	*0.13*	*84.1*	*0.707*	*0.122*	*0.3790*	*0.2664*	*0.003890*	*0.001870*	*0.000619*	*0.001870*	*3.99*	*12.05*	
KRB07-33	*30μmPE0*	*204*	*366*	*0.56*	*0.11*	*86.0*	*0.722*	*0.153*	*0.4399*	*0.5098*	*0.004420*	*0.004856*	*0.000618*	*0.004856*	*3.98*	*31.23*	
KRB07-34	30μmPE0	13,090	4,372	2.99	0.60	5.4	0.089	0.010	0.0019	0.0002	0.000154	0.000004	0.000146	0.000004	0.94	0.03	
KRB07-35	*30μmPE0*	*303*	*403*	*0.75*	*0.15*	*65.3*	*0.559*	*0.040*	*0.0349*	*0.0067*	*0.000453*	*0.000079*	*0.000157*	*0.000079*	*1.01*	*0.51*	
KRB07-36	30μmPE0	1,065	1,750	0.61	0.12	-0.5	0.042	0.001	0.0565	0.0027	0.009694	0.000425	0.009694	0.000425	62.19	2.74	
KRB07-37	*30μmPE0*	*72*	*135*	*0.53*	*0.11*	*42.0*	*0.376*	*0.055*	*0.1274*	*0.0447*	*0.002455*	*0.000578*	*0.001423*	*0.000578*	*9.17*	*3.72*	
KRB07-38	30μmPE0	105	230	0.46	0.09	39.3	0.355	0.057	0.0422	0.0051	0.000862	0.000111	0.000523	0.000111	3.37	0.72	
KRB07-39	30μmPE0	179	281	0.64	0.13	0.8	0.052	0.004	0.0731	0.0052	0.010163	0.000482	0.010084	0.000482	64.68	3.11	
KRB07-40	30μmPE0	415	699	0.59	0.12	27.7	0.264	0.069	0.0074	0.0018	0.000203	0.000014	0.000147	0.000014	0.95	0.09	
KRB07-41	*30μmPE0*	*146*	*287*	*0.51*	*0.10*	*53.5*	*0.467*	*0.065*	*0.0280*	*0.0041*	*0.000435*	*0.000061*	*0.000202*	*0.000061*	*1.30*	*0.40*	
KRB07-42	*30μmPE0*	*340*	*475*	*0.72*	*0.14*	*68.3*	*0.583*	*0.062*	*0.1525*	*0.0177*	*0.001900*	*0.000273*	*0.000603*	*0.000273*	*3.89*	*1.76*	
KRB07-43	30μmPE0	193	358	0.54	0.11	0.6	0.051	0.004	0.0676	0.0036	0.009657	0.000307	0.009600	0.000307	61.59	1.98	
KRB07-44	30μmPE0	1,020	864	1.18	0.24	13.5	0.152	0.046	0.0086	0.0032	0.000410	0.000041	0.000354	0.000041	2.28	0.27	
KRB07-45	30μmPE0	218	264	0.82	0.16	0.5	0.050	0.004	0.0630	0.0062	0.009128	0.000761	0.009081	0.000761	58.28	4.90	
KRB07-46	*30μmPE0*	*249*	*230*	*1.08*	*0.22*	*73.5*	*0.623*	*0.144*	*0.1215*	*0.0627*	*0.001414*	*0.000542*	*0.000375*	*0.000542*	*2.42*	*3.49*	
KRB07-47	*30μmPE0*	*166*	*264*	*0.63*	*0.13*	*64.5*	*0.553*	*0.052*	*0.0934*	*0.0326*	*0.001226*	*0.000367*	*0.000436*	*0.000367*	*2.81*	*2.37*	
KRB07-48	30μmPE0	439	365	1.20	0.24	19.7	0.201	0.028	0.0130	0.0023	0.000468	0.000034	0.000375	0.000034	2.42	0.22	
KRB07-49	*30μmPE0*	*221*	*255*	*0.87*	*0.17*	*82.5*	*0.694*	*0.056*	*0.9084*	*0.4815*	*0.009494*	*0.004862*	*0.001664*	*0.004862*	*10.72*	*31.27*	
KRB07-50	30μmPE0	249	251	0.99	0.20	2.2	0.063	0.011	0.0804	0.0175	0.009237	0.000490	0.009036	0.000490	57.99	3.16	
KRB07-51	30μmPE5	404	437	0.92	0.18	9.0	0.117	0.045	0.0064	0.0018	0.000395	0.000040	0.000359	0.000040	2.31	0.26	
KRB07-52	30μmPE5	387	326	1.19	0.24	25.7	0.248	0.075	0.0075	0.0019	0.000220	0.000046	0.000164	0.000046	1.05	0.30	
KRB07-53	*30μmPE5*	*252*	*349*	*0.72*	*0.14*	*43.2*	*0.385*	*0.066*	*0.0402*	*0.0065*	*0.000756*	*0.000142*	*0.000430*	*0.000142*	*2.77*	*0.92*	
KRB07-54	30μmPE5	230	293	0.78	0.16	32.9	0.305	0.098	0.0190	0.0060	0.000452	0.000020	0.000304	0.000020	1.96	0.13	
KRB07-55	30μmPE5	218	222	0.98	0.20	0.0	0.046	0.004	0.0564	0.0061	0.008847	0.000590	0.008845	0.000590	56.77	3.80	
KRB07-56	30μmPE5	726	552	1.32	0.26	50.3	0.441	0.047	0.0238	0.0052	0.000391	0.000054	0.000194	0.000054	1.25	0.35	
KRB07-57	*30μmPE5*	*343*	*444*	*0.77*	*0.15*	*9.2*	*0.119*	*0.121*	*0.0103*	*0.0025*	*0.000630*	*0.000457*	*0.000572*	*0.000457*	*3.69*	*2.94*	
KRB07-58	30μmPE5	210	244	0.86	0.17	16.3	0.174	0.071	0.0051	0.0023	0.000214	0.000027	0.000179	0.000027	1.15	0.18	
KRB07-59	*30μmPE5*	*277*	*287*	*0.97*	*0.19*	*54.7*	*0.476*	*0.114*	*0.0319*	*0.0123*	*0.000486*	*0.000093*	*0.000220*	*0.000093*	*1.42*	*0.60*	
KRB07-60	30μmPE5	385	527	0.73	0.15	13.1	0.149	0.022	0.0083	0.0013	0.000402	0.000022	0.000350	0.000022	2.25	0.14	
KRB07-61	*30μmPE5*	*303*	*254*	*1.19*	*0.24*	*140.1*	*1.147*	*1.476*	*0.6774*	*0.6704*	*0.004284*	*0.001139*	*-0.001719*	*0.001139*	*-11.09*	*7.34*	
KRB07-62	30μmPE5	1,082	1,261	0.86	0.17	6.2	0.094	0.013	0.0049	0.0006	0.000373	0.000012	0.000350	0.000012	2.26	0.08	
KRB07-63	30μmPE5	3,370	1,890	1.78	0.36	4.5	0.081	0.017	0.0042	0.0008	0.000374	0.000017	0.000357	0.000017	2.30	0.11	
KRB07-64	30μmPE5	305	388	0.79	0.16	27.3	0.261	0.307	0.0153	0.0139	0.000424	0.000075	0.000309	0.000075	1.99	0.49	
KRB07-65	*30μmPE5*	*280*	*294*	*0.95*	*0.19*	*69.3*	*0.591*	*0.279*	*0.0583*	*0.0274*	*0.000716*	*0.000193*	*0.000220*	*0.000193*	*1.42*	*1.24*	
KRB07-66	*30μmPE5*	*866*	*186*	*4.65*	*0.93*	*74.7*	*0.633*	*0.107*	*0.2263*	*0.0558*	*0.002594*	*0.000452*	*0.000657*	*0.000452*	*4.24*	*2.91*	
KRB07-67	30μmPE5	1,273	1,168	1.09	0.22	0.3	0.049	0.003	0.0624	0.0060	0.009279	0.000380	0.009247	0.000380	59.34	2.45	
KRB07-68	30μmPE5	244	325	0.75	0.15	3.0	0.070	0.020	0.0065	0.0022	0.000676	0.000061	0.000656	0.000061	4.23	0.39	
KRB07-69	30μmPE5	725	851	0.85	0.17	8.6	0.114	0.020	0.0060	0.0014	0.000381	0.000034	0.000349	0.000034	2.25	0.22	
KRB07-70	30μmPE5	841	707	1.19	0.24	5.2	0.087	0.021	0.0049	0.0010	0.000410	0.000058	0.000389	0.000058	2.51	0.38	
KRB07-71	30μmPE5	642	646	0.99	0.20	11.6	0.137	0.030	0.0092	0.0022	0.000487	0.000025	0.000431	0.000025	2.78	0.16	
KRB07-72	30μmPE5	881	1,845	0.48	0.10	0.0	0.046	0.001	0.0625	0.0019	0.009763	0.000298	0.009758	0.000298	62.60	1.92	
KRB07-73	30μmPE5	429	378	1.14	0.23	9.2	0.119	0.027	0.0131	0.0030	0.000804	0.000056	0.000730	0.000056	4.70	0.36	
KRB07-74	30μmPE5	645	631	1.02	0.20	29.3	0.276	0.074	0.0189	0.0067	0.000496	0.000040	0.000351	0.000040	2.26	0.26	
KRB07-75	*30μmPE5*	*142*	*215*	*0.66*	*0.13*	*59.1*	*0.511*	*0.065*	*0.0659*	*0.0146*	*0.000936*	*0.000122*	*0.000383*	*0.000122*	*2.47*	*0.79*	
KRB07-76	*30μmPE5*	*118*	*117*	*1.01*	*0.20*	*6.4*	*0.096*	*0.013*	*0.0349*	*0.0114*	*0.002640*	*0.000799*	*0.002472*	*0.000799*	*15.92*	*5.15*	
KRB07-77	30μmPE5	328	353	0.93	0.19	5.0	0.085	0.034	0.0015	0.0006	0.000129	0.000008	0.000123	0.000008	0.79	0.05	
KRB07-78	30μmPE5	118	175	0.67	0.13	11.7	0.138	0.048	0.0076	0.0026	0.000401	0.000049	0.000354	0.000049	2.28	0.32	
KRB07-79	30μmPE5	235	235	1.00	0.20	30.7	0.287	0.075	0.0211	0.0086	0.000533	0.000106	0.000369	0.000106	2.38	0.68	
KRB07-80	30μmPE5	306	364	0.84	0.17	19.5	0.199	0.051	0.0139	0.0044	0.000505	0.000047	0.000407	0.000047	2.62	0.30	
KRB07-81	*30μmPE5*	*194*	*142*	*1.36*	*0.27*	*32.7*	*0.303*	*0.072*	*0.0204*	*0.0034*	*0.000488*	*0.000115*	*0.000329*	*0.000115*	*2.12*	*0.74*	
KRB07-82	30μmPE5	1,034	811	1.27	0.25	2.5	0.066	0.012	0.0037	0.0007	0.000411	0.000027	0.000400	0.000027	2.58	0.17	
KRB07-83	*30μmPE5*	*486*	*391*	*1.24*	*0.25*	*22.3*	*0.221*	*0.038*	*0.0134*	*0.0041*	*0.000441*	*0.000133*	*0.000343*	*0.000133*	*2.21*	*0.86*	
KRB07-84	30μmPE5	1,339	2,314	0.58	0.12	-0.3	0.044	0.001	0.0577	0.0035	0.009503	0.000621	0.009503	0.000621	60.97	4.00	

Sample name	Condition[a]	Th	U	Th/U	f^{b}	f_{206}%[c]	Total						Radiogenic		Age [Ma]		MSWD[d]
		(ppm)	(ppm)				$^{207}Pb/^{206}Pb$	2σ	$^{207}Pb/^{235}U$	2σ	$^{206}Pb/^{238}U$	2σ	$^{206}Pb/^{238}U$	2σ	$^{206}Pb/^{238}U$	2σ	
KRB07-85	30μmPE5	727	1,492	0.49	0.10	-0.5	0.042	0.002	0.0534	0.0021	0.009140	0.000431	0.009140	0.000431	58.65	2.78	
KRB07-86	30μmPE5	207	345	0.60	0.12	8.6	0.114	0.029	0.0145	0.0038	0.000926	0.000170	0.000847	0.000170	5.45	1.10	
KRB07-87	30μmPE5	788	877	0.90	0.18	8.4	0.112	0.037	0.0055	0.0011	0.000358	0.000075	0.000328	0.000075	2.12	0.48	
KRB07-88	30μmPE5	595	510	1.17	0.23	20.7	0.209	0.130	0.0108	0.0051	0.000376	0.000108	0.000298	0.000108	1.92	0.70	
KRB07-89	30μmPE5	255	423	0.60	0.12	49.4	0.434	0.302	0.0097	0.0068	0.000163	0.000026	0.000082	0.000026	0.53	0.17	
KRB07-90	30μmPE5	245	236	1.04	0.21	49.6	0.436	0.142	0.0402	0.0175	0.000669	0.000135	0.000337	0.000135	2.17	0.87	
KRB07-91	30μmPE5	377	409	0.92	0.18	42.4	0.380	0.121	0.0432	0.0245	0.000826	0.000228	0.000476	0.000228	3.07	1.47	
KRB07-92	30μmPE5	62	118	0.53	0.11	32.9	0.304	0.133	0.0445	0.0177	0.001060	0.000278	0.000712	0.000278	4.59	1.79	
KRB07-93	30μmPE5	480	549	0.87	0.17	25.9	0.250	0.115	0.0065	0.0046	0.000190	0.000056	0.000140	0.000056	0.91	0.36	
KRB07-94	30μmPE5	550	357	1.54	0.31	15.2	0.166	0.046	0.0117	0.0045	0.000511	0.000057	0.000433	0.000057	2.79	0.37	
KRB07-95	30μmPE5	364	247	1.47	0.29	25.7	0.248	0.079	0.0125	0.0029	0.000365	0.000395	0.000271	0.000395	1.75	2.55	
KRB07-96	30μmPE5	810	962	0.84	0.17	2.7	0.067	0.012	0.0033	0.0006	0.000352	0.000018	0.000342	0.000018	2.21	0.11	
KRB07-97	30μmPE5	617	701	0.88	0.18	10.0	0.124	0.037	0.0042	0.0014	0.000245	0.000012	0.000221	0.000012	1.42	0.08	
KRB07-98	30μmPE5	119	161	0.74	0.15	32.9	0.305	0.059	0.0225	0.0042	0.000536	0.000066	0.000360	0.000066	2.32	0.43	
KRB07-99	30μmPE5	208	178	1.17	0.23	29.5	0.278	0.123	0.0190	0.0078	0.000495	0.000058	0.000349	0.000058	2.25	0.38	
KRB07-100	30μmPE5	140	208	0.67	0.13	19.4	0.199	0.052	0.0148	0.0047	0.000539	0.000104	0.000434	0.000104	2.80	0.67	
KRB07-2-1	30μmPE20	564	723	0.78	0.16	86.8	0.728	1.511	0.3055	0.2049	0.003044	0.001050	0.000402	0.001050	2.59	6.77	
KRB07-2-2	30μmPE20	610	1,135	0.54	0.11	4.6	0.082	0.011	0.1195	0.0179	0.010566	0.000417	0.010082	0.000417	64.67	2.69	
KRB07-2-3	30μmPE20	571	566	1.01	0.20	66.6	0.569	0.195	0.2141	0.1175	0.002728	0.000789	0.000911	0.000789	5.87	5.08	
KRB07-2-4	30μmPE20	2,295	1,928	1.19	0.24	41.1	0.369	0.072	0.0854	0.0220	0.001681	0.000351	0.000990	0.000351	6.38	2.26	
KRB07-2-5	30μmPE20	1,076	2,022	0.53	0.11	32.1	0.299	0.187	0.0268	0.0158	0.000651	0.000077	0.000442	0.000077	2.85	0.49	
KRB07-2-6	30μmPE20	4,666	2,017	2.31	0.46	46.3	0.410	0.085	0.0562	0.0177	0.000994	0.000197	0.000534	0.000197	3.44	1.27	
KRB07-2-7	30μmPE20	569	578	0.98	0.20	47.4	0.419	0.197	0.0977	0.0554	0.001691	0.000969	0.000889	0.000969	5.73	6.25	
KRB07-2-8	30μmPE20	241	245	0.98	0.20	72.8	0.618	0.322	0.2232	0.1291	0.002621	0.000582	0.000713	0.000582	4.60	3.75	
KRB07-2-9	30μmPE20	348	283	1.23	0.25	101.6	0.844	0.614	1.6229	1.1913	0.013944	0.001117	-0.000222	0.001117	-1.43	7.20	
KRB07-2-10	30μmPE20	309	380	0.81	0.16	97.1	0.809	0.246	1.3986	0.4775	0.012540	0.004318	0.000362	0.004318	2.33	27.78	
KRB07-2-11	30μmPE20	161	158	1.02	0.20	109.0	0.903	1.499	0.9664	0.4241	0.007766	0.004684	-0.000701	0.004684	-4.52	30.13	
KRB07-2-12	30μmPE20	111	239	0.47	0.09	109.6	0.907	0.217	4.0215	0.7234	0.032160	0.007811	-0.003082	0.007811	-19.90	50.16	
KRB07-2-13	30μmPE20	218	459	0.47	0.09	-0.7	0.040	0.061	0.0098	0.0045	0.001759	0.005575	0.001759	0.005575	11.33	35.84	
KRB07-2-13(2)	30μmPE20	218	459	0.47	0.09	6.1	0.094	0.061	0.0098	0.0045	0.000755	0.000119	0.000709	0.000119	4.57	0.77	
KRB07-2-14	30μmPE20	215	212	1.02	0.20	32.6	0.303	0.131	0.0205	0.0066	0.000492	0.000213	0.000332	0.000213	2.14	1.37	
KRB07-2-15	30μmPE20	210	171	1.23	0.25	16.6	0.177	0.415	0.2070	0.0844	0.008493	0.010133	0.007080	0.010133	45.48	64.99	
KRB07-2-15(2)	30μmPE20	210	171	1.23	0.25	40.7	0.366	0.400	0.2070	0.0844	0.004106	0.001792	0.002435	0.001792	15.68	11.54	
KRB07-2-16	30μmPE20	79	106	0.75	0.15	56.6	0.491	0.213	0.0569	0.0442	0.000841	0.000238	0.000365	0.000238	2.36	1.53	
KRB07-2-17	30μmPE20	200	244	0.82	0.16	177.1	1.438	2.384	0.2093	0.3001	0.001056	0.000343	-0.000814	0.000343	-5.25	2.21	
KRB07-2-18	30μmPE20	180	347	0.52	0.10	27.6	0.263	0.087	0.0169	0.0066	0.000466	0.000047	0.000337	0.000047	2.17	0.30	
KRB07-2-19	30μmPE20	121	138	0.88	0.18	2.2	0.064	0.012	0.0835	0.0200	0.009535	0.001222	0.009323	0.001222	59.82	7.87	
KRB07-2-20	30μmPE20	209	291	0.72	0.14	36.2	0.330	0.072	0.0306	0.0094	0.000673	0.000113	0.000430	0.000113	2.77	0.73	
KRB07-2-21	30μmPE20	764	290	2.63	0.53	75.1	0.637	0.129	2.2252	0.7201	0.025360	0.006131	0.006303	0.006131	40.50	39.40	
KRB07-2-22	30μmPE20	497	529	0.94	0.19	65.5	0.561	0.128	0.1481	0.0427	0.001916	0.000461	0.000661	0.000461	4.26	2.97	
Azusa River (sampling location coordinates: 36.249975,137.637266)																	
JP3-1	30μmUE0	152	80	1.89	0.38	13.9	0.155	0.102	0.0064	0.0033	0.000300	0.000047	0.000259	0.000047	1.67	0.30	
JP3-2	30μmUE0	737	497	1.48	0.30	4.6	0.082	0.017	0.0029	0.0006	0.000258	0.000014	0.000246	0.000014	1.59	0.09	
JP3-3	30μmUE0	120	170	0.70	0.14	-0.7	0.041	0.003	0.0586	0.0047	0.010381	0.000383	0.010381	0.000383	66.57	2.47	
JP3-4	30μmUE0	103	189	0.54	0.11	0.6	0.051	0.004	0.0659	0.0119	0.009367	0.001750	0.009308	0.001750	59.73	11.27	
JP3-5	30μmUE0	140	78	1.79	0.36	6.4	0.096	0.111	0.0043	0.0038	0.000321	0.000045	0.000300	0.000045	1.94	0.29	
JP3-6	30μmUE0	151	125	1.21	0.24	-0.5	0.042	0.003	0.0642	0.0065	0.011078	0.000952	0.011078	0.000952	71.02	6.13	
JP3-7	30μmUE0	1,483	611	2.43	0.49	4.9	0.084	0.028	0.0035	0.0011	0.000304	0.000020	0.000289	0.000020	1.86	0.13	
JP3-8	30μmUE0	125	86	1.47	0.29	3.2	0.072	0.009	0.0992	0.0158	0.010052	0.000523	0.009725	0.000523	62.39	3.37	
JP3-9	30μmUE0	311	313	1.00	0.20	-0.3	0.044	0.005	0.0591	0.0061	0.009845	0.000643	0.009845	0.000643	63.15	4.15	
JP3-10	30μmUE0	515	607	0.85	0.17	-0.5	0.043	0.002	0.0544	0.0037	0.009278	0.000421	0.009278	0.000421	59.53	2.71	
JP3-11	30μmUE0	685	596	1.15	0.23	4.1	0.078	0.027	0.0028	0.0010	0.000258	0.000026	0.000248	0.000026	1.60	0.17	
JP3-12	30μmUE0	186	114	1.63	0.33	0.7	0.052	0.005	0.0916	0.0115	0.012826	0.001166	0.012732	0.001166	81.56	7.51	
JP3-13	30μmUE0	1,196	1,371	0.87	0.17	-0.5	0.042	0.002	0.0587	0.0038	0.010074	0.000382	0.010074	0.000382	64.62	2.46	
JP3-14	30μmUE0	139	150	0.93	0.19	-0.3	0.043	0.005	0.0593	0.0065	0.009917	0.000446	0.009917	0.000446	63.62	2.87	
JP3-15	30μmUE0	174	131	1.33	0.27	7.9	0.108	0.056	0.0075	0.0031	0.000504	0.000107	0.000465	0.000107	2.99	0.69	
JP3-16	30μmUE0	142	471	0.30	0.06	7.0	0.101	0.002	4.5147	0.3399	0.323426	0.024441	0.300716	0.024441	1694.86	155.66	
JP3-17	30μmUE0	429	581	0.74	0.15	-0.5	0.042	0.002	0.0590	0.0049	0.010152	0.000630	0.010152	0.000630	65.11	4.06	
JP3-18	30μmUE0	791	1,729	0.46	0.09	-0.3	0.043	0.002	0.0648	0.0046	0.010820	0.000242	0.010820	0.000242	69.38	1.56	
JP3-19	30μmUE0	175	251	0.70	0.14	0.5	0.050	0.007	0.0739	0.0090	0.010729	0.000250	0.010675	0.000250	68.45	1.61	
JP3-20	30μmUE0	957	1,169	0.82	0.16	-0.6	0.041	0.002	0.0592	0.0025	0.010380	0.000326	0.010380	0.000326	66.57	2.10	
JP3-21	30μmUE0	415	576	0.72	0.14	-0.6	0.041	0.002	0.0600	0.0050	0.010530	0.000661	0.010530	0.000661	67.52	4.26	
JP3-22	30μmUE0	300	388	0.77	0.15	9.1	0.118	0.050	0.0096	0.0054	0.000590	0.000471	0.000536	0.000471	3.46	3.03	
JP3-23	30μmUE0	1,164	1,005	1.16	0.23	-0.4	0.043	0.001	0.0637	0.0044	0.010792	0.000539	0.010792	0.000539	69.19	3.48	
JP3-24	30μmUE0	133	223	0.60	0.12	0.3	0.048	0.005	0.0715	0.0075	0.010756	0.000529	0.010727	0.000529	68.79	3.41	
JP3-25	30μmUE0	218	467	0.47	0.09	-0.2	0.045	0.002	0.0351	0.0104	0.005699	0.001632	0.005699	0.001632	36.64	10.51	
JP3-26	30μmUE0	583	511	1.14	0.23	9.9	0.124	0.037	0.0040	0.0015	0.000237	0.000020	0.000213	0.000020	1.37	0.13	
JP3-27	30μmUE0	1,023	996	1.03	0.21	34.1	0.314	0.061	0.0160	0.0032	0.000369	0.000024	0.000243	0.000024	1.57	0.15	
JP3-28	30μmUE0	1,074	2,316	0.46	0.09	-0.4	0.043	0.001	0.0508	0.0056	0.008517	0.000799	0.008517	0.000799	54.67	5.15	
JP3-29	30μmUE0	125	190	0.66	0.13	-0.2	0.044	0.007	0.0580	0.0086	0.009517	0.000275	0.009517	0.000275	61.06	1.77	
JP3-30	30μmUE0	994	1,349	0.74	0.15	-0.5	0.042	0.001	0.0559	0.0021	0.009686	0.000113	0.009686	0.000113	62.14	0.73	
JP3-31	30μmUE0	100	240	0.41	0.08	6.9	0.101	0.018	0.0118	0.0023	0.000849	0.000046	0.000790	0.000046	5.09	0.30	
JP3-32	30μmUE0	245	820	0.30	0.06	4.2	0.079	0.013	0.0030	0.0005	0.000272	0.000015	0.000260	0.000015	1.68	0.10	
JP3-33	30μmUE0	217	359	0.60	0.12	-0.1	0.045	0.003	0.0653	0.0039	0.010540	0.000292	0.010540	0.000292	67.59	1.88	
JP3-34	30μmUE0	128	187	0.69	0.14	28.9	0.273	0.658	0.0018	0.0016	0.000047	0.000017	0.000033	0.000017	0.21	0.11	
JP3-35	30μmUE0	212	331	0.64	0.13	-0.3	0.044	0.005	0.0555	0.0050	0.009154	0.000415	0.009154	0.000415	58.75	2.68	
JP3-36	30μmUE0	322	695	0.46	0.09	0.0	0.046	0.003	0.0537	0.0061	0.008465	0.000624	0.008465	0.000624	54.34	4.02	
JP3-37	30μmUE0	624	831	0.75	0.15	-0.6	0.041	0.002	0.0554	0.0037	0.009772	0.000565	0.009772	0.000565	62.69	3.64	
JP3-38	30μmUE0	83	63	1.32	0.26	3.3	0.072	0.058	0.0132	0.0091	0.001329	0.000215	0.001284	0.000215	8.28	1.39	
JP3-39	30μmUE0	137	195	0.70	0.14	6.7	0.099	0.089	0.0039	0.0031	0.000286	0.000039	0.000267	0.000039	1.72	0.25	
JP3-40	30μmUE0	264	534	0.49	0.10	-0.6	0.042	0.002	0.0548	0.0049	0.009565	0.000587	0.009565	0.000587	61.37	3.79	
JP3-41	30μmUE0	123	263	0.47	0.09	-0.4	0.043	0.004	0.0555	0.0068	0.009354	0.001096	0.009354	0.001096	60.02	7.06	
JP3-42	30μmUE0	408	875	0.47	0.09	-0.5	0.042	0.002	0.0635	0.0047	0.010939	0.000727	0.010939	0.000727	70.14	4.68	
JP3-43	30μmUE0	203	158	1.29	0.26	19.4	0.198	0.107	0.0123	0.0058	0.000451	0.000094	0.000364	0.000094	2.35	0.61	
JP3-44	30μmUE0	1,966	3,088	0.64	0.13	-0.8	0.040	0.001	0.0600	0.0018	0.010899	0.000545	0.010899	0.000545	69.88	3.51	
JP3-45	30μmUE0	466	560	0.83	0.17	0.2	0.047	0.021	0.0018	0.0008	0.000280	0.000030	0.000279	0.000030	1.80	0.20	
JP3-46	30μmUE0	174	143	1.22	0.24	24.4	0.237	0.083	0.0125	0.0043	0.000382	0.000097	0.000289	0.000097	1.86	0.63	

Sample name	Condition[a]	Th (ppm)	U (ppm)	Th/U	f^{b}	f_{206}%[c]	Total $^{207}Pb/^{206}Pb$	2σ	$^{207}Pb/^{235}U$	2σ	$^{206}Pb/^{238}U$	2σ	Radiogenic $^{206}Pb/^{238}U$	2σ	Age [Ma] $^{206}Pb/^{238}U$	2σ	MSWD[d]
JP3-47	30μmUE0	888	1,859	0.48	0.10	-0.4	0.043	0.002	0.0631	0.0050	0.010649	0.000614	0.010649	0.000614	68.29	3.96	
JP3-48	30μmUE0	128	246	0.52	0.10	7.6	0.106	0.044	0.0060	0.0025	0.000414	0.000051	0.000383	0.000051	2.47	0.33	
JP3-49	30μmUE0	89	87	1.02	0.20	-0.7	0.040	0.006	0.0554	0.0105	0.009974	0.000435	0.009974	0.000435	63.98	2.81	
JP3-50	30μmUE0	80	106	0.75	0.15	8.3	0.111	0.094	0.0041	0.0036	0.000265	0.000042	0.000243	0.000042	1.56	0.27	
JP3-51	30μmPE13	871	894	0.97	0.19	14.0	0.156	0.106	0.0080	0.0043	0.000372	0.000082	0.000320	0.000082	2.07	0.53	
JP3-52	30μmPE13	450	525	0.86	0.17	76.2	0.645	0.483	0.0799	0.0431	0.000900	0.000330	0.000214	0.000330	1.38	2.12	
JP3-53	30μmPE13	546	677	0.81	0.16	3.9	0.077	0.061	0.1996	0.0979	0.018886	0.013679	0.018151	0.013679	115.96	87.58	
JP3-53(2)	30μmPE13	546	677	0.81	0.16	9.9	0.124	0.062	0.1996	0.0979	0.011690	0.001009	0.010533	0.001009	67.55	6.50	
JP3-54	30μmPE13	2,389	1,225	1.95	0.39	14.4	0.159	0.031	0.2864	0.0747	0.013069	0.001078	0.011191	0.001078	71.74	6.95	
JP3-55	30μmPE13	648	447	1.45	0.29	1.7	0.059	0.004	0.0901	0.0068	0.011026	0.000425	0.010841	0.000425	69.51	2.74	
JP3-56	30μmPE13	2,254	380	5.92	1.18	26.1	0.251	0.072	0.5373	0.2172	0.015534	0.002945	0.011484	0.002945	73.61	18.96	
JP3-57	30μmPE13	3,131	1,500	2.09	0.42	-0.3	0.044	0.002	0.0650	0.0034	0.010760	0.000426	0.010760	0.000426	68.99	2.75	
JP3-58	30μmPE13	234	217	1.08	0.22	19.0	0.195	0.044	0.0104	0.0024	0.000386	0.000044	0.000313	0.000044	2.01	0.28	
JP3-59	30μmPE13	92	82	1.12	0.22	32.8	0.304	0.462	0.5329	0.7321	0.012712	0.001283	0.008538	0.001283	54.81	8.26	
JP3-60	30μmPE13	358	223	1.61	0.32	30.4	0.285	0.095	0.8750	0.5300	0.022257	0.009087	0.015484	0.009087	99.05	58.31	
JP3-61	30μmPE13	234	323	0.72	0.14	4.4	0.080	0.027	0.1294	0.0529	0.011688	0.001236	0.011178	0.001236	71.66	7.97	
JP3-62	30μmPE13	148	152	0.97	0.19	46.9	0.415	0.447	0.0434	0.0247	0.000759	0.000552	0.000403	0.000552	2.60	3.55	
JP3-63	30μmPE13	291	224	1.30	0.26	65.5	0.561	0.217	0.4667	0.2892	0.006038	0.003339	0.002084	0.003339	13.42	21.49	
JP3-64	30μmPE13	1,314	1,205	1.09	0.22	143.8	1.176	0.997	0.3060	0.2688	0.001888	0.001259	-0.000827	0.001259	-5.33	8.11	
JP3-65	30μmPE13	380	375	1.01	0.20	7.1	0.102	0.046	0.1626	0.0725	0.011592	0.001444	0.010771	0.001444	69.06	9.30	
JP3-66	30μmPE13	468	353	1.33	0.27	16.2	0.173	0.078	0.3334	0.2502	0.013976	0.002462	0.011718	0.002462	75.10	15.85	
JP3-67	30μmPE13	501	461	1.09	0.22	66.2	0.567	0.293	0.1515	0.0464	0.001941	0.000944	0.000655	0.000944	4.22	6.08	
JP3-68	30μmPE13	112	129	0.86	0.17	11.2	0.134	0.087	0.2357	0.1422	0.012735	0.001488	0.011305	0.001488	72.47	9.59	
JP3-69	30μmPE13	271	220	1.23	0.25	108.4	0.898	1.659	0.1977	0.1344	0.001598	0.000957	-0.000134	0.000957	-0.87	6.16	
JP3-70	30μmPE13	286	243	1.18	0.24	63.8	0.547	0.931	0.0946	0.0850	0.001253	0.000518	0.000454	0.000518	2.93	3.34	
JP3-71	30μmPE13	456	351	1.30	0.26	1.3	0.056	0.017	0.0817	0.0229	0.010559	0.000760	0.010425	0.000760	66.85	4.90	
JP3-72	30μmPE13	267	228	1.17	0.23	44.0	0.392	0.198	0.0786	0.0390	0.001456	0.000466	0.000816	0.000466	5.26	3.00	
JP3-73	30μmPE13	254	254	1.00	0.20	53.2	0.464	0.369	0.1064	0.0521	0.001663	0.000582	0.000779	0.000582	5.02	3.75	
JP3-74	30μmPE13	629	536	1.17	0.23	1.1	0.055	0.006	0.0786	0.0108	0.010348	0.000483	0.010229	0.000483	65.61	3.11	
JP3-2-1	30μmPE20	70	63	1.11	0.22	0.2	0.048	0.008	0.0657	0.0112	0.010037	0.000347	0.010019	0.000347	64.27	2.24	
JP3-2-2	30μmPE20	283	202	1.40	0.28	43.5	0.388	0.058	0.0371	0.0126	0.000693	0.000156	0.000391	0.000156	2.52	1.00	
JP3-2-3	30μmPE20	324	470	0.69	0.14	21.5	0.215	0.039	0.0090	0.0019	0.000304	0.000023	0.000239	0.000023	1.54	0.15	
JP3-2-4	30μmPE20	219	183	1.20	0.24	55.1	0.479	0.608	0.1984	0.1066	0.003003	0.001297	0.001347	0.001297	8.68	8.36	
JP3-2-5	30μmPE20	191	193	0.99	0.20	86.7	0.728	0.534	0.0677	0.0677	0.000675	0.000236	0.000090	0.000236	0.58	1.52	
JP3-2-6	30μmPE20	454	474	0.96	0.19	85.8	0.720	0.052	0.8329	0.3691	0.008393	0.003590	0.001195	0.003590	7.70	23.10	
JP3-2-7	30μmPE20	623	314	1.98	0.40	57.8	0.500	0.146	0.2871	0.0774	0.004164	0.001288	0.001758	0.001288	11.32	8.30	
JP3-2-8	30μmPE20	195	140	1.40	0.28	73.9	0.627	1.153	0.1188	0.0759	0.001375	0.001636	0.000359	0.001636	2.32	10.54	
JP3-2-9	30μmPE20	342	228	1.50	0.30	0.2	0.048	0.269	0.0278	0.0146	0.004219	0.006338	0.004210	0.006338	27.08	40.73	
JP3-2-9(2)	30μmPE20	342	228	1.50	0.30	13.9	0.155	0.265	0.0278	0.0146	0.001299	0.000687	0.001119	0.000687	7.21	4.42	
JP3-2-10	30μmPE20	436	352	1.24	0.25	3.3	0.072	0.024	0.1099	0.0348	0.011024	0.000568	0.010656	0.000568	68.33	3.66	
JP3-2-11	30μmPE20	237	314	0.75	0.15	2.7	0.067	0.045	0.1058	0.0553	0.011451	0.001725	0.011146	0.001725	71.45	11.11	
JP3-2-12	30μmPE20	5,121	1,898	2.70	0.54	11.6	0.137	0.064	0.0086	0.0021	0.000454	0.000287	0.000401	0.000287	2.58	1.85	
JP3-2-13	30μmPE20	575	566	1.02	0.20	37.0	0.337	0.187	0.0614	0.0360	0.001322	0.000554	0.000833	0.000554	5.37	3.57	
JP3-2-14	30μmPE20	187	190	0.99	0.20	83.6	0.703	0.849	0.1740	0.2122	0.001796	0.000579	0.000295	0.000579	1.90	3.73	
JP3-2-15	30μmPE20	269	397	0.68	0.14	24.3	0.237	0.053	0.0102	0.0028	0.000311	0.000029	0.000235	0.000029	1.52	0.19	
JP3-2-16	30μmPE20	422	664	0.64	0.13	11.3	0.135	0.181	0.0064	0.0064	0.000347	0.000168	0.000308	0.000168	1.99	1.09	
JP3-2-17	30μmPE20	281	177	1.59	0.32	1.1	0.055	0.011	0.0835	0.0128	0.011070	0.001188	0.010948	0.001188	70.19	7.65	
JP3-2-18	30μmPE20	519	216	2.40	0.48	43.0	0.384	0.290	0.7880	0.2212	0.014891	0.008329	0.008489	0.008329	54.49	53.47	
JP3-2-19	30μmPE20	698	531	1.31	0.26	70.0	0.596	0.472	0.0996	0.0147	0.001213	0.000290	0.000364	0.000290	2.35	1.87	
JP3-2-20	30μmPE20	416	530	0.78	0.16	14.4	0.159	0.384	0.0260	0.0151	0.001184	0.001428	0.001013	0.001428	6.53	9.20	
JP3-2-20(2)	30μmPE20	416	530	0.78	0.16	34.7	0.319	0.375	0.0260	0.0151	0.000592	0.000201	0.000386	0.000201	2.49	1.29	
JP3-2-21	30μmPE20	187	187	1.00	0.20	33.9	0.312	1.246	0.1419	0.1182	0.003299	0.003559	0.002182	0.003559	14.05	22.90	
JP3-2-21(2)	30μmPE20	187	187	1.00	0.20	56.8	0.493	1.238	0.1419	0.1182	0.002090	0.001527	0.000902	0.001527	5.81	9.84	
JP3-2-22	30μmPE20	167	179	0.93	0.19	102.0	0.848	2.181	0.1925	0.1413	0.001648	0.000745	-0.000033	0.000745	-0.21	4.80	
JP3-2-23	30μmPE20	340	335	1.02	0.20	85.9	0.721	0.700	0.1220	0.1270	0.001227	0.000293	0.000173	0.000293	1.12	1.89	
JP3-2-24	30μmPE20	212	204	1.04	0.21	111.2	0.920	4.079	0.2135	0.2300	0.001684	0.000886	-0.000188	0.000886	-1.21	5.71	
JP3-2-25	30μmPE20	40	100	0.40	0.08	14.1	0.157	0.055	0.2508	0.0950	0.011576	0.000757	0.009940	0.000757	63.76	4.88	
JP3-2-26	30μmPE20	277	276	1.00	0.20	73.6	0.625	0.685	0.1349	0.0801	0.001566	0.000461	0.000413	0.000461	2.66	2.97	
JP3-2-27	30μmPE20	139	140	0.99	0.20	-0.4	0.043	0.003	0.0571	0.0040	0.009573	0.000328	0.009573	0.000328	61.42	2.11	
JP3-2-28	30μmPE20	418	579	0.72	0.14	36.7	0.334	0.851	0.0434	0.0440	0.000941	0.000509	0.000596	0.000509	3.84	3.28	
JP3-2-29	30μmPE20	346	442	0.78	0.16	2.1	0.062	0.024	0.0891	0.0322	0.010354	0.000272	0.010139	0.000272	65.03	1.75	
JP3-2-30	30μmPE20	221	254	0.87	0.17	47.3	0.418	0.340	0.0906	0.0250	0.001573	0.000602	0.000829	0.000602	5.34	3.88	
JP3-2-31	30μmPE20	695	498	1.40	0.28	7.8	0.108	0.038	0.2013	0.0650	0.013580	0.001919	0.012518	0.001919	80.20	12.36	
JP3-2-32	30μmPE20	277	175	1.58	0.32	48.4	0.427	1.178	0.1282	0.1476	0.002179	0.001307	0.001124	0.001307	7.24	8.42	
JP3-2-33	30μmPE20	185	169	1.09	0.22	57.2	0.496	0.276	0.1207	0.1244	0.001765	0.001322	0.000755	0.001322	4.86	8.51	
JP3-2-34	30μmPE20	275	583	0.47	0.09	1.6	0.059	0.009	0.0815	0.0163	0.010041	0.000576	0.009877	0.000576	63.36	3.71	
JP3-2-35	30μmPE20	493	331	1.49	0.30	81.7	0.688	0.537	1.3743	0.6149	0.014493	0.006596	0.002655	0.006596	17.09	42.38	
JP3-2-36	30μmPE20	380	438	0.87	0.17	46.0	0.407	0.253	0.0338	0.0150	0.000602	0.000147	0.000325	0.000147	2.10	0.95	
JP3-2-37	30μmPE20	258	102	2.52	0.50	94.2	0.786	0.746	2.5695	1.2328	0.023708	0.010612	0.001376	0.010612	8.86	68.05	
JP3-2-38	30μmPE20	594	190	3.14	0.63	22.8	0.226	0.051	0.4927	0.1221	0.015848	0.002809	0.012229	0.002809	78.35	18.08	
JP3-2-39	30μmPE20	339	551	0.61	0.12	9.4	0.120	0.045	0.0039	0.0016	0.000238	0.000014	0.000216	0.000014	1.39	0.09	
JP3-2-40	30μmPE20	1,023	1,761	0.58	0.12	2.5	0.066	0.013	0.0891	0.0182	0.009790	0.000272	0.009542	0.000272	61.22	1.75	
JP3-2-41	30μmPE20	151	190	0.79	0.16	7.8	0.107	0.102	0.0073	0.0068	0.000491	0.000266	0.000452	0.000266	2.92	1.71	
JP3-2-42	30μmPE20	182	282	0.64	0.13	22.1	0.219	0.100	0.4346	0.3217	0.014370	0.006842	0.011200	0.006842	71.80	43.96	
JP3-2-43	30μmPE20	164	208	0.79	0.16	41.1	0.369	1.527	0.0699	0.0604	0.001374	0.001361	0.000809	0.001361	5.21	8.76	
JP3-2-44	30μmPE20	103	85	1.21	0.24	191.2	1.549	1.110	0.8461	1.0532	0.003964	0.001186	-0.003615	0.001186	-23.35	7.64	
JP3-2-44(2)	30μmPE20	103	85	1.21	0.24	99.3	0.826	0.720	0.4502	0.1942	0.003954	0.001186	0.000029	0.001186	0.19	7.64	
JP3-2-101	30μmPE20	170	169	1.00	0.20	2.8	0.068	0.057	0.0988	0.0761	0.010578	0.000226	0.010287	0.000226	65.97	1.46	
JP3-2-102	30μmPE20	253	338	0.75	0.15	4.5	0.082	0.039	0.1264	0.0528	0.011223	0.000856	0.010714	0.000856	68.70	5.52	
JP3-2-103	30μmPE20	264	442	0.60	0.12	-0.4	0.043	0.008	0.0700	0.0097	0.011802	0.002044	0.011802	0.002044	75.64	13.16	
JP3-2-104	30μmPE20	133	113	1.18	0.24	14.7	0.162	0.139	0.7479	0.6279	0.033552	0.001053	0.028615	0.001053	181.88	6.79	
JP3-2-105	30μmPE20	1,454	1,435	1.01	0.20	3.4	0.073	0.014	0.1156	0.0205	0.011485	0.000401	0.011092	0.000401	71.11	2.58	
JP3-2-106	30μmPE20	607	830	0.73	0.15	3.6	0.075	0.017	0.1181	0.0251	0.011485	0.000316	0.011068	0.000316	70.96	2.04	
JP3-2-107	30μmPE20	1,061	1,429	0.74	0.15	13.5	0.152	0.066	0.2740	0.1019	0.013084	0.001922	0.011321	0.001922	72.57	12.38	
JP3-2-108	30μmPE20	942	1,331	0.71	0.14	-0.1	0.045	0.001	0.0622	0.0028	0.010027	0.000434	0.010027	0.000434	64.32	2.80	
JP3-2-109	30μmPE20	122	178	0.68	0.14	4.5	0.082	0.034	0.1201	0.0607	0.010664	0.001022	0.010180	0.001022	65.29	6.58	
JP3-2-110	30μmPE20	398	350	1.14	0.23	16.2	0.173	0.023	0.4147	0.0404	0.017370	0.001566	0.014560	0.001566	93.18	10.08	

Sample name	Condition[a]	Th (ppm)	U (ppm)	Th/U	f^b	f_{206}%[c]	Total ^{207}Pb/^{206}Pb	2σ	^{207}Pb/^{235}U	2σ	^{206}Pb/^{238}U	2σ	Radiogenic ^{206}Pb/^{238}U	2σ	Age [Ma] ^{206}Pb/^{238}U	2σ	MSWD[d]
Kurobegawa Granite (felsic part) (sampling location coordinates: 36.698894,137.681243)																	
KRB02-F-1	40μmUE0	362,238	2,730	132.69	26.54	68.8	0.587	0.110	0.1173	0.0406	0.001450	0.000434	0.000452	0.000434	2.92	2.80	
KRB02-F-2	40μmUE0	583	1,582	0.37	0.07	54.9	0.478	0.746	0.0115	0.0163	0.000175	0.000010	0.000079	0.000010	0.51	0.06	
KRB02-F-3	40μmUE0	233	667	0.35	0.07	56.1	0.487	0.089	0.0197	0.0054	0.000293	0.000058	0.000129	0.000058	0.83	0.37	
KRB02-F-4	40μmUE0	3,192	5,090	0.63	0.13	6.6	0.098	0.010	0.0021	0.0002	0.000152	0.000009	0.000142	0.000009	0.91	0.06	
KRB02-F-5	40μmUE0	906	1,123	0.81	0.16	12.0	0.141	0.054	0.0033	0.0012	0.000169	0.000008	0.000149	0.000008	0.96	0.05	
KRB02-F-6	40μmUE0	458	847	0.54	0.11	28.1	0.267	0.216	0.0058	0.0048	0.000157	0.000034	0.000113	0.000034	0.73	0.22	
KRB02-F-7	40μmUE0	423	1,240	0.34	0.07	27.8	0.264	0.173	0.0056	0.0034	0.000153	0.000013	0.000111	0.000013	0.71	0.08	
KRB02-F-8	40μmUE0	231	587	0.39	0.08	29.6	0.279	0.088	0.0063	0.0022	0.000164	0.000017	0.000115	0.000017	0.74	0.11	
KRB02-F-9	40μmUE0	3,577	3,875	0.92	0.18	27.4	0.262	0.063	0.0071	0.0030	0.000196	0.000035	0.000142	0.000035	0.92	0.23	
KRB02-F-10	40μmUE0	1,213	2,155	0.56	0.11	19.9	0.202	0.060	0.0047	0.0014	0.000167	0.000009	0.000134	0.000009	0.86	0.06	
KRB02-F-11	30μmUE0	122	193	0.63	0.13	60.3	0.520	0.125	0.0928	0.0283	0.001295	0.000555	0.000514	0.000555	3.31	3.58	
KRB02-F-12	30μmUE0	243	314	0.78	0.16	62.5	0.538	0.204	0.0196	0.0123	0.000264	0.000042	0.000099	0.000042	0.64	0.27	
KRB02-F-13	30μmUE0	193	280	0.69	0.14	44.4	0.395	0.161	0.0104	0.0045	0.000191	0.000014	0.000106	0.000014	0.68	0.09	
KRB02-F-14	30μmUE0	411	503	0.82	0.16	41.0	0.368	0.043	0.0124	0.0028	0.000245	0.000043	0.000144	0.000043	0.93	0.28	
KRB02-F-15	30μmUE0	162	234	0.69	0.14	1046	8.265	18.770	0.3078	0.6056	0.000270	0.000048	-0.002556	0.000048	-16.50	0.31	
KRB02-F-15(2)	30μmUE0	162	234	0.69	0.14	53.7	0.468	0.082	0.0167	0.0034	0.000259	0.000048	0.000120	0.000048	0.77	0.31	
KRB02-F-16	30μmUE0	180	322	0.56	0.11	38.5	0.349	0.120	0.0101	0.0045	0.000209	0.000037	0.000129	0.000037	0.83	0.24	
KRB02-F-17	30μmUE0	202	355	0.57	0.11	12.8	0.147	0.116	0.0029	0.0017	0.000144	0.000026	0.000126	0.000026	0.81	0.16	
KRB02-F-18	30μmUE0	264	405	0.65	0.13	34.3	0.316	0.074	0.0079	0.0018	0.000181	0.000029	0.000119	0.000029	0.77	0.19	
KRB02-F-19	30μmUE0	182	336	0.54	0.11	86.7	0.727	0.444	0.0245	0.0210	0.000245	0.000043	0.000033	0.000043	0.21	0.28	
KRB02-F-20	30μmUE0	170	306	0.56	0.11	59.3	0.512	0.103	0.0201	0.0061	0.000284	0.000040	0.000116	0.000040	0.75	0.26	
													**Weighted mean without no. 1, 2, 3, 4, 5, 6, 11, 12, 15, 15(2), 19, 20**		**0.78**	**0.04**	**2.3**
Kurobegawa Granite (mafic part or MMEs) (sampling location coordinates: 36.698894,137.681243)																	
KRB02-M-1	40μmUE0	149	396	0.38	0.08	55.5	0.482	0.538	0.0096	0.0148	0.000144	0.000014	0.000064	0.000014	0.41	0.09	
KRB02-M-2	40μmUE0	166	409	0.41	0.08	13.7	0.154	0.038	0.0029	0.0007	0.000139	0.000013	0.000120	0.000013	0.77	0.08	
KRB02-M-3	40μmUE0	374	845	0.44	0.09	5.7	0.091	0.035	0.0018	0.0008	0.000146	0.000009	0.000138	0.000009	0.89	0.06	
KRB02-M-4	40μmUE0	392	745	0.53	0.11	28.9	0.274	0.402	0.0052	0.0070	0.000139	0.000013	0.000099	0.000013	0.64	0.09	
KRB02-M-5	40μmUE0	603	1,090	0.55	0.11	23.7	0.232	0.226	0.0047	0.0043	0.000148	0.000004	0.000113	0.000004	0.73	0.03	
KRB02-M-6	40μmUE0	391	699	0.56	0.11	12.0	0.140	0.018	0.0029	0.0004	0.000152	0.000007	0.000134	0.000007	0.86	0.04	
KRB02-M-7	40μmUE0	386	882	0.44	0.09	12.5	0.144	0.020	0.0030	0.0006	0.000151	0.000016	0.000132	0.000016	0.85	0.10	
KRB02-M-8	40μmUE0	477	813	0.59	0.12	11.1	0.134	0.141	0.0033	0.0026	0.000182	0.000093	0.000162	0.000093	1.04	0.60	
KRB02-M-9	40μmUE0	264	421	0.63	0.13	12.0	0.140	0.038	0.0023	0.0007	0.000121	0.000015	0.000107	0.000015	0.69	0.10	
KRB02-M-10	40μmUE0	218	935	0.23	0.05	0.1	0.047	0.044	0.0066	0.0016	0.001022	0.000348	0.001021	0.000348	6.58	2.24	
KRB02-M-11	30μmUE0	230	381	0.60	0.12	14.1	0.157	0.053	0.0030	0.0008	0.000139	0.000018	0.000119	0.000018	0.77	0.11	
KRB02-M-12	30μmUE0	231	424	0.54	0.11	11.9	0.140	0.041	0.0038	0.0011	0.000195	0.000016	0.000171	0.000016	1.10	0.10	
KRB02-M-13	30μmUE0	297	345	0.86	0.17	51.2	0.448	0.095	0.0260	0.0118	0.000421	0.000134	0.000205	0.000134	1.32	0.86	
KRB02-M-14	30μmUE0	315	439	0.72	0.14	11.0	0.133	0.033	0.0026	0.0007	0.000140	0.000009	0.000124	0.000009	0.80	0.06	
KRB02-M-15	30μmUE0	359	441	0.81	0.16	8.3	0.111	0.037	0.0020	0.0006	0.000129	0.000014	0.000118	0.000014	0.76	0.09	
KRB02-M-16	30μmUE0	1,120	838	1.34	0.27	1.9	0.061	0.029	0.0012	0.0006	0.000145	0.000011	0.000143	0.000011	0.92	0.07	
KRB02-M-17	30μmUE0	283	325	0.87	0.17	8.2	0.111	0.100	0.0028	0.0015	0.000185	0.000021	0.000170	0.000021	1.09	0.14	
KRB02-M-18	30μmUE0	242	276	0.87	0.17	17.5	0.183	0.083	0.0043	0.0014	0.000171	0.000039	0.000141	0.000039	0.91	0.25	
KRB02-M-19	30μmUE0	504	587	0.86	0.17	10.5	0.129	0.062	0.0033	0.0013	0.000185	0.000016	0.000165	0.000016	1.06	0.10	
KRB02-M-20	30μmUE0	331	284	1.17	0.23	20.9	0.211	0.074	0.0057	0.0017	0.000197	0.000027	0.000155	0.000027	1.00	0.17	
													**Weighted mean without no. 1, 3, 4, 8, 10, 12, 13, 16, 17, 19, 20**		**0.75**	**0.02**	**1.7**
Takidani Granodiorite (sampling location coordinates: 36.249733,137.637566)																	
JP4-1	30μmUE0	353	434	0.81	0.16	6.3	0.095	0.095	0.0200	0.0045	0.001520	0.000948	0.001425	0.000948	9.18	6.11	
JP4-2	30μmUE0	549	633	0.87	0.17	19.7	0.201	0.055	0.0081	0.0023	0.000294	0.000033	0.000236	0.000033	1.52	0.21	
JP4-3	30μmUE0	430	516	0.83	0.17	10.0	0.125	0.024	0.0046	0.0007	0.000267	0.000024	0.000240	0.000024	1.55	0.16	
JP4-4	30μmUE0	265	410	0.65	0.13	71.3	0.606	0.062	0.1079	0.0313	0.001292	0.000318	0.000371	0.000318	2.39	2.05	
JP4-5	30μmUE0	431	469	0.92	0.18	14.2	0.158	0.029	0.0064	0.0015	0.000294	0.000028	0.000252	0.000028	1.62	0.18	
JP4-6	30μmUE0	262	423	0.62	0.12	5.8	0.091	0.027	0.0032	0.0011	0.000254	0.000023	0.000239	0.000023	1.54	0.15	
JP4-7	30μmUE0	315	411	0.77	0.15	35.4	0.324	0.047	0.0188	0.0030	0.000420	0.000035	0.000271	0.000035	1.75	0.22	
JP4-8	30μmUE0	275	392	0.70	0.14	33.2	0.307	0.047	0.0157	0.0028	0.000371	0.000035	0.000248	0.000035	1.60	0.23	
JP4-9	30μmUE0	306	374	0.82	0.16	30.1	0.283	0.066	0.0196	0.0029	0.000503	0.000155	0.000351	0.000155	2.27	1.00	
JP4-10	30μmUE0	398	493	0.81	0.16	31.3	0.292	0.030	0.0138	0.0018	0.000343	0.000032	0.000236	0.000032	1.52	0.21	
JP4-11	30μmUE0	410	472	0.87	0.17	22.4	0.222	0.024	0.0098	0.0006	0.000319	0.000018	0.000248	0.000018	1.60	0.12	
JP4-12	30μmUE0	318	452	0.70	0.14	10.4	0.128	0.032	0.0041	0.0009	0.000235	0.000017	0.000211	0.000017	1.36	0.11	
JP4-13	30μmUE0	2,106	1,835	1.15	0.23	3.2	0.071	0.011	0.0023	0.0005	0.000238	0.000021	0.000231	0.000021	1.49	0.13	
JP4-14	30μmUE0	251	282	0.89	0.18	11.0	0.133	0.042	0.0052	0.0017	0.000286	0.000023	0.000255	0.000023	1.64	0.15	
JP4-15	30μmUE0	346	490	0.71	0.14	35.3	0.323	0.063	0.0182	0.0044	0.000409	0.000032	0.000265	0.000032	1.71	0.21	
JP4-16	30μmUE0	281	297	0.95	0.19	36.6	0.334	0.043	0.0175	0.0015	0.000381	0.000035	0.000241	0.000035	1.56	0.22	
JP4-17	30μmUE0	1,412	1,364	1.03	0.21	13.7	0.154	0.016	0.0058	0.0006	0.000276	0.000016	0.000238	0.000016	1.53	0.10	
JP4-18	30μmUE0	143	280	0.51	0.10	6.6	0.098	0.024	0.0039	0.0010	0.000291	0.000031	0.000272	0.000031	1.75	0.20	
JP4-19	30μmUE0	648	697	0.93	0.19	23.9	0.234	0.146	0.0081	0.0051	0.000250	0.000020	0.000191	0.000020	1.23	0.13	
JP4-20	30μmUE0	518	574	0.90	0.18	49.5	0.435	0.030	0.0308	0.0028	0.000513	0.000048	0.000259	0.000048	1.67	0.31	
													**Weighted mean without no. 1, 4, 9, 12, 19**		**1.58**	**0.04**	**0.9**
OD-3 (reference age: 33.0 ± 0.1 Ma; Iwano et al., 2013)																	
LAB14-2-11	30μmUE0	477	382	1.25		-1.4	0.035	0.005	0.0244	0.0030	0.005050	0.000193	0.005050	0.000193	32.5	1.2	
LAB14-2-12	30μmPE0	745	552	1.35		0.4	0.049	0.007	0.0358	0.0063	0.005267	0.000223	0.005245	0.000223	33.7	1.4	
LAB14-2-13	30μmUE0	214	196	1.09		-1.2	0.036	0.005	0.0264	0.0032	0.005242	0.000192	0.005242	0.000192	33.7	1.2	
LAB14-2-14	30μmUE0	377	315	1.20		-1.5	0.034	0.004	0.0249	0.0028	0.005260	0.000212	0.005260	0.000212	33.8	1.4	
LAB14-2-15	30μmUE0	212	214	0.99		-1.5	0.034	0.007	0.0228	0.0044	0.004852	0.000443	0.004852	0.000443	31.2	2.9	
LAB14-2-16	30μmUE0	417	348	1.20		-0.9	0.039	0.006	0.0282	0.0031	0.005223	0.000289	0.005223	0.000289	33.6	1.9	
LAB14-2-17	30μmUE0	1,240	943	1.32		-1.0	0.038	0.003	0.0272	0.0027	0.005130	0.000267	0.005130	0.000267	33.0	1.7	
LAB14-2-18	30μmUE0	1,330	943	1.41		-1.2	0.037	0.003	0.0268	0.0027	0.005268	0.000253	0.005268	0.000253	33.9	1.6	
LAB14-2-19	30μmUE0	532	442	1.20		-1.3	0.036	0.004	0.0269	0.0029	0.005414	0.000288	0.005414	0.000288	34.8	1.9	
LAB14-2-20	30μmUE0	984	759	1.30		-1.3	0.036	0.002	0.0260	0.0021	0.005261	0.000301	0.005261	0.000301	33.8	1.9	
													**Weighted mean**		**33.5**	**0.5**	**0.9**
OD-3 (reference age: 33.0 ± 0.1 Ma; Iwano et al., 2013)																	
LAB14-2-11E	30μmPE13	789	575	1.37		7.5	0.105	0.050	0.0770	0.0361	0.005313	0.000301	0.004914	0.000301	31.6	1.9	
LAB14-2-12E	30μmPE13	779	536	1.45		52.2	0.456	0.132	0.7827	0.3860	0.012447	0.003891	0.005950	0.003891	38.2	25.0	
LAB14-2-13E	30μmPE13	469	324	1.45		1.2	0.055	0.008	0.0406	0.0057	0.005324	0.000124	0.005262	0.000124	33.8	0.8	
LAB14-2-14E	30μmPE13	583	424	1.38		3.3	0.072	0.029	0.0514	0.0204	0.005180	0.000138	0.005009	0.000138	32.2	0.9	

Sample name	Condition[a]	Th	U	Th/U	f[b]	f_{206}%[c]	Total						Radiogenic		Age [Ma]		MSWD[d]
		(ppm)	(ppm)				$^{207}Pb/^{206}Pb$	2σ	$^{207}Pb/^{235}U$	2σ	$^{206}Pb/^{238}U$	2σ	$^{206}Pb/^{238}U$	2σ	$^{206}Pb/^{238}U$	2σ	
LAB14-2-15E	30μmPE13	561	416	1.35		4.5	0.082	0.020	0.0642	0.0317	0.005698	0.000938	0.005440	0.000938	35.0	6.0	
LAB14-2-16E	30μmPE13	241	208	1.15		3.2	0.072	0.013	0.0524	0.0081	0.005308	0.000199	0.005136	0.000199	33.0	1.3	
LAB14-2-17E	30μmPE13	928	621	1.49		0.8	0.052	0.006	0.0375	0.0046	0.005231	0.000074	0.005192	0.000074	33.4	0.5	
LAB14-2-18E	*30μmPE13*	*1,305*	*534*	*2.44*		*28.4*	*0.269*	*0.141*	*0.3086*	*0.1357*	*0.008320*	*0.001928*	*0.005959*	*0.001928*	*38.3*	*12.4*	
LAB14-2-19E	30μmPE13	707	498	1.42		1.6	0.059	0.003	0.0454	0.0027	0.005620	0.000174	0.005530	0.000174	35.6	1.1	
LAB14-2-20E	30μmPE13	519	387	1.34		0.0	0.046	0.003	0.0335	0.0021	0.005293	0.000102	0.005293	0.000102	34.0	0.7	
													**Weighted mean without no. 12, 18**		**33.5**	**0.3**	**4.4**
Plesovice (reference age: 337.13 ± 0.37 Ma; Slama et al., 2008)																	
P1-1-3	40μmPE0	247	1,855	0.13		0.0	0.046	0.000	0.3152	0.0192	0.049420	0.003076	0.049410	0.003076	310.9	19.8	
P1-1-4	40μmPE0	208	1,528	0.14		-0.1	0.045	0.000	0.3375	0.0068	0.053993	0.001191	0.053993	0.001191	339.0	7.7	
P1-1-5	40μmPE0	427	3,327	0.13		-0.1	0.045	0.000	0.3702	0.0070	0.059126	0.001030	0.059126	0.001030	370.3	6.6	
P1-1-6	40μmPE0	217	2,058	0.11		-0.1	0.046	0.000	0.3643	0.0047	0.057943	0.000765	0.057943	0.000765	363.1	4.9	
P3-1-60	40μmPE0	140	1,033	0.08		-2.2	0.029	0.000	0.2159	0.0029	0.054277	0.001038	0.054277	0.001038	340.7	6.7	
P3-1-61	40μmPE0	120	899	0.07		-2.2	0.029	0.001	0.2087	0.0061	0.053027	0.001185	0.053027	0.001185	333.1	7.6	
P3-1-264	30μmPE0	102	797	0.13		-0.6	0.041	0.002	0.3127	0.0144	0.055130	0.001411	0.055130	0.001411	345.9	9.1	
P3-1-265	30μmPE0	85	690	0.12		-0.6	0.041	0.001	0.3224	0.0087	0.056790	0.000654	0.056790	0.000654	356.1	4.2	
P3-1-296	30μmPE0	126	799	0.16		0.1	0.047	0.001	0.3434	0.0102	0.052991	0.000765	0.052929	0.000765	332.5	4.9	
P3-1-297	30μmPE0	113	586	0.19		1.2	0.056	0.003	0.4414	0.0211	0.057500	0.001269	0.056798	0.001269	356.1	8.2	
P3-1-298	30μmPE0	117	832	0.14		0.1	0.047	0.001	0.3600	0.0074	0.055332	0.001105	0.055255	0.001105	346.7	7.1	
P3-1-299	30μmPE0	139	1,140	0.12		0.3	0.049	0.001	0.3471	0.0173	0.051820	0.001767	0.051655	0.001767	324.7	11.4	
P3-1-300	30μmPE0	116	941	0.12		0.3	0.048	0.001	0.3737	0.0105	0.055985	0.001003	0.055819	0.001003	350.1	6.5	
P3-1-301	30μmPE0	208	1,123	0.19		0.7	0.051	0.001	0.3731	0.0079	0.052775	0.000823	0.052426	0.000823	329.4	5.3	
P3-1-302	30μmPE0	218	1,182	0.18		0.2	0.047	0.001	0.3552	0.0097	0.054414	0.001093	0.054327	0.001093	341.0	7.0	
P3-1-303	30μmPE0	119	937	0.13		0.3	0.048	0.001	0.3625	0.0129	0.054424	0.001939	0.054269	0.001939	340.7	12.5	
P3-1-304	30μmPE0	158	1,014	0.16		5.4	0.089	0.006	0.7083	0.0544	0.057825	0.001920	0.054678	0.001920	343.2	12.4	
P3-1-305	30μmPE0	155	1,059	0.15		0.3	0.048	0.002	0.3575	0.0146	0.053898	0.000627	0.053758	0.000627	337.6	4.0	
P3-1-315	30μmPE0	101	889	0.11		0.2	0.048	0.001	0.3595	0.0102	0.054637	0.001029	0.054522	0.001029	342.2	6.6	
P3-1-316	30μmPE0	126	992	0.13		0.3	0.049	0.001	0.3553	0.0152	0.053108	0.001532	0.052943	0.001532	332.6	9.9	
P3-1-317	30μmPE0	102	797	0.13		0.2	0.048	0.001	0.3623	0.0091	0.055106	0.001018	0.054993	0.001018	345.1	6.6	
P3-1-327	30μmPE0	140	1,142	0.12		0.5	0.050	0.001	0.3494	0.0138	0.051064	0.001901	0.050833	0.001901	319.6	12.2	
P3-1-328	30μmPE0	146	1,104	0.13		0.3	0.048	0.002	0.3514	0.0105	0.052889	0.000693	0.052747	0.000693	331.4	4.5	
P3-1-329	30μmPE0	155	1,118	0.14		0.3	0.048	0.001	0.3426	0.0090	0.051632	0.001067	0.051498	0.001067	323.7	6.9	
P3-1-332	30μmPE0	126	1,043	0.12		0.2	0.047	0.001	0.3346	0.0057	0.051282	0.000846	0.051201	0.000846	321.9	5.5	
P3-1-333	30μmPE0	112	923	0.12		0.2	0.048	0.002	0.3440	0.0091	0.052238	0.000968	0.052126	0.000968	327.6	6.2	
P3-1-334	30μmPE0	140	1,090	0.13		0.2	0.048	0.002	0.3316	0.0074	0.050514	0.001377	0.050415	0.001377	317.1	8.9	
P3-1-335	30μmPE0	102	862	0.12		0.1	0.047	0.001	0.3165	0.0089	0.048747	0.001190	0.048685	0.001190	306.4	7.7	
P3-1-336	30μmPE0	97	809	0.12		0.3	0.048	0.001	0.3237	0.0140	0.048449	0.001978	0.048303	0.001978	304.1	12.7	
P3-1-337	30μmPE0	92	756	0.12		0.1	0.047	0.001	0.3417	0.0101	0.052980	0.000845	0.052933	0.000845	332.5	5.4	
P3-1-338	30μmPE0	174	1,360	0.13		0.3	0.048	0.001	0.3753	0.0129	0.056297	0.001529	0.056135	0.001529	352.1	9.9	
P3-1-339	30μmPE0	121	1,079	0.11		0.9	0.053	0.002	0.4146	0.0257	0.056594	0.003649	0.056085	0.003649	351.8	23.5	
P3-1-340	30μmPE0	106	963	0.11		0.6	0.051	0.002	0.4066	0.0225	0.057717	0.002260	0.057348	0.002260	359.5	14.6	
P3-1-341	30μmPE0	287	1,965	0.15		1.0	0.054	0.002	0.3845	0.0232	0.051720	0.001440	0.051204	0.001440	321.9	9.3	
P3-1-342	30μmPE0	132	1,089	0.12		4.4	0.081	0.003	0.6391	0.0153	0.057205	0.001435	0.054660	0.001435	343.1	9.2	
P3-1-343	30μmPE0	116	989	0.12		0.4	0.049	0.001	0.3658	0.0171	0.054302	0.001821	0.054110	0.001821	339.7	11.7	
P3-1-344	30μmPE0	238	1,729	0.14		1.0	0.054	0.002	0.3879	0.0177	0.052181	0.001660	0.051661	0.001660	324.7	10.7	
P3-1-345	30μmPE0	186	1,561	0.12		0.4	0.050	0.001	0.3539	0.0181	0.051839	0.002575	0.051612	0.002575	324.4	16.6	
P3-1-346	30μmPE0	187	1,351	0.14		4.3	0.080	0.003	0.6023	0.0285	0.054513	0.001399	0.052150	0.001399	327.7	9.0	
P3-1-347	30μmPE0	205	1,390	0.15		4.1	0.078	0.008	0.5799	0.0740	0.053898	0.001648	0.051705	0.001648	325.0	10.6	
P3-1-348	30μmPE0	185	1,264	0.15		0.5	0.050	0.001	0.3626	0.0111	0.052794	0.001181	0.052544	0.001181	330.1	7.6	
P3-1-349	30μmPE0	203	1,477	0.14		0.4	0.049	0.002	0.3634	0.0103	0.053578	0.000979	0.053365	0.000979	335.1	6.3	
P3-1-350	30μmPE0	153	1,120	0.14		0.3	0.048	0.001	0.3493	0.0112	0.052595	0.000876	0.052456	0.000876	329.6	5.6	
P3-1-351	30μmPE0	172	1,212	0.14		2.2	0.064	0.010	0.4842	0.0862	0.055103	0.001446	0.053865	0.001446	338.2	9.3	
													**Weighted mean without no. 5, 335, 336**		**337.7**	**1.1**	**10.8**

[a] Laser beam diameter (40μm or 30μm), polished or unpolished (P or U), eticng time (E0 for unetched, E20 for 20-hour-etched for instance) are shown.

[b] $f = (Th/U)_{zircon}/(Th/U)_{magma}$. Samples except for standards were calculated assuming initial ^{230}Th disequilibrium using f value. $(Th/U)_{magma}$ of 5.0 was used.

[c] f_{206}% denotes the percentage of ^{206}Pb that is common Pb. Correction for common Pb was made using measured $^{206}Pb/^{238}U$ and $^{207}Pb/^{206}Pb$ ratios. Data calculated less than 0% were treated as 0%.

[d] MSWD: mean square weighted deviation.
